# Metabolomic and Genomic Analysis of Bioactive Compounds of *Phacidium infestans* Karsten DSM 5139 Cultivated on *Pinus sylvestris* Needles

**DOI:** 10.1111/1758-2229.70084

**Published:** 2025-06-09

**Authors:** Chahira Zerouki, Omolara Mofikoya, Taskeen Badar, Marko Mäkinen, Ossi Turunen, Janne Jänis

**Affiliations:** ^1^ School of Forest Sciences University of Eastern Finland Joensuu Finland; ^2^ Department of Environmental and Biological Sciences University of Eastern Finland Joensuu Finland; ^3^ Department of Chemistry and Sustainable Technology University of Eastern Finland Joensuu Finland

**Keywords:** FT‐ICR, *Gremmenia infestans*, mass spectrometry, needles, *Phacidium infestans Karsten* DSM 5139, *Pinus sylvestris*, secreted carbohydrate‐active enzymes

## Abstract

This study investigates how *Phacidium infestans* acquires nutrients on *Pinus sylvestris* needles, which possess antimicrobial properties. *P. infestans* was evaluated for its growth and enzyme production on various substrates, alongside genomic and metabolomic analysis. Direct‐infusion high‐resolution mass spectrometry (DI‐HRMS) was performed on methanol extracts obtained from *P. infestans* cultivated on needles and malt extract media. DI‐HRMS analysis identified 21 compounds from the malt extract and 112 from the needle samples. The resin components increased in the needle samples post‐cultivation, suggesting terpenoid release from resin ducts due to fungal degradation of plant cell walls. *P. infestans* fully consumed sugars and antifungal compounds, including taxiresinol and salicylic acid, with control‐to‐sample ratios (CTR/SA) of 289.76 and 47.24, respectively. Moreover, lariciresinol and pinoresinol were reduced to undetectable levels. The genomic analysis identified 421 secreted proteins, including 128 carbohydrate‐active enzymes, 3 cutinases, and 49 lipases that aid host penetration and wax degradation. Several multi‐drug efflux pumps and two acyclic terpene utilisation proteins were identified as well. These proteins support the cellular integrity of *P. infestans* by expelling toxic compounds. Our findings provide valuable insights into the metabolic strategies of *P. infestans* for nutrient assimilation on pine needles.

## Graphical abstract

*Phacidium infestans* DSM 5139 was studied for its ability to penetrate pine needles and overcome their inhibitory compounds. Using mass spectrometry and genome mining, we identified the biological and molecular strategies enabling nutrient acquisition and resilience against chemical stress, providing insights into the survival mechanisms of this cold‐adapted fungal pathogen.

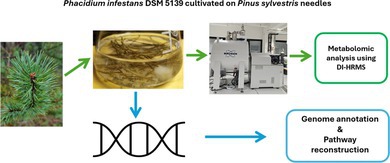

## 1 Background

*Phacidium infestans* (also known as *Gremmenia infestans*) is one of the main diseases of coniferous trees (Lilja et al. 2010). This fungus is the causative agent of snow blight, and it is widely distributed in northern regions of Eurasia and North America. The needles of all ages, buds and bark are killed by this fungus under the snow cover (Roll‐Hansen 1989). In addition to the resilience of *P. infestans* to freezing temperatures (Zerouki et al. 2023), this fungus thrives on pine needles, which contain five times more terpenes than the wood (Manninen et al. 2002).

The needles from various pine tree species contain a variety of compounds that have been shown to exhibit antimicrobial properties (Dziedziński et al. 2021). Terpenes and their derivatives are among the diverse chemical compounds synthesised by pine trees. Their specific composition compounds can vary between species, environmental conditions, and other factors (Kännaste et al. 2013). Terpenes are well known for their antimicrobial and bioactive properties, enabling pine trees to survive and adapt to their environment. They play a significant role in protecting pine trees against herbivores and pathogenic microorganisms (Jung et al. 2007; Ji and Ji 2021; Kim et al. 2020; Kopaczyk et al. 2020; Mahizan et al. 2019; Zulak and Bohlmann 2010). In addition, *Pinus* species, and pine needles in particular, have considerable potential as a source of medicinal applications (Kim et al. 2013). Previous research on pine needle extracts has shown that they possess analgesic, anti‐inflammatory, sedative, antitussive, expectorant, antipruritic, antiasthmatic, antioxidant, antimutation, lipid regulating and antitumor activities (Wang et al. 2014).

The current research aims to investigate strategies employed by *P. infestans* to survive on the pine needles. Due to their chemical composition, enriched by compounds such as terpenes, resins and other secondary metabolites, the needles represent a unique challenge for the growth of microorganisms. In fact, the fungus must first successfully penetrate the waxy surface of the needles and overcome the inhibitory effects of the needle's constituents. Moreover, *P. infestans* needs to acquire sufficient nutrients in such hostile environments. In this study, a direct‐infusion high‐resolution mass spectrometry (DI‐HRMS) analysis was performed on methanol extracts obtained by cultivating *P. infestans* on pine ( *Pinus sylvestris* ) needles and malt extract (control). Two complementary ionisation techniques, namely, negative‐ion electrospray ionisation (ESI) and positive‐ion atmospheric pressure photoionization (APPI), were employed to target both polar and non‐polar metabolites, respectively (Mofikoya et al. 2020). *P. infestans* was further studied by analysing its relevant genomic features and metabolic behaviour. The ability of the fungus to degrade pine needle cell wall components was assessed by cultivating it on various substrates and conducting enzyme screening, supported by genomic annotation of its secreted carbohydrate‐active enzymes (CAZymes).

Understanding the biological and molecular strategies employed by *P. infestans* to thrive in adverse conditions is crucial to uncovering its ecological adaptations. This study is one of the first metabolomic and genomic investigations of *P. infestans* cultivated on pine needles and aims to provide deeper insights into the resilience of this cold‐adapted fungus.

## 2 Materials and Methods

### 2.1 Cultural Characterisation and Extracellular Enzymes

The strain *P. infestans* Karst. DSM 5139 was purchased from the DSMZ collection. The fungus was initially cultivated on Malt Extract Agar (MEA) plates (2% malt extract and 2% agar, pH 5.9) and incubated at 22°C until sufficient growth was obtained. Agar plugs (6 mm) were made on the MEA plates that contained the fungus. The surface of the plugs was scraped to transfer equal amounts of fungal mycelium with no MEA medium to new cultivation plates.

#### 2.1.1 Preferential Carbon Sources

The preferential carbon source of the fungus was evaluated on minimal media (MM), pH 5.9, (g/L): KH_2_PO_4_, 0.68; MgSO_4_·7H_2_O, 0.1; NaCl, 4.0; FeSO_4_·7H_2_O, 0.03; NH_4_NO_3_, 1.2; CaCl_2_·2H_2_O, 0.02. This medium was supplemented with 1% of different carbon sources (carboxymethylcellulose (CMC), pectin, xylan, lignin, or starch) (Sati and Bisht 2006). MEA plates were used as a control.

The growth of the fungus was recorded by measuring the radius of the colony in two directions. The measurement was performed from the centre of the colony to the external edges of the mycelial growth. The radial growth bioassays were stopped when the fungal mycelia reached the Petri dish wall. Five replicates were used for each experiment.

#### 2.1.2 Screening for Extracellular Enzymes

Cellulolytic activity was screened by inoculating the fungus onto the CMC agar medium (pH 5.9) that contained 1% carboxymethyl cellulose (CMC) (Admassie et al. 2022). The medium composition was as follows (g/L): NH_4_H_2_PO_4_, 1.0; KCl, 0.2; MgSO_4_·7H_2_O, 1.0; yeast extract, 1.0; CMC, 10; agar, 20. After 5 days of incubation at 22°C, the CMC agar plates were flooded with Congo red for 10 min and then washed with 1 M NaCl at room temperature. The clear zone diameter of CMC hydrolysis to the colony diameter was measured and recorded for all the plates (Florencio et al. 2012).

Pectinase screening was assayed using the following medium (g/L): NaNO_3_, 1.0; KCl, 1.0; K_2_HPO_4_, 1.0; MgSO_4_, 0.5; pectin, 10; agar, 20; pH 5.9. After the inoculation of the fungus, the plates were incubated at 22°C for 5 days. Identification of enzyme activity was performed by flooding the plate with iodine solution (Kalaichelvan 2012).

To detect the xylanase production ability, the fungus was inoculated on 1% xylan agar medium (g/L); yeast extract, 3.0; peptone, 1.5; KH_2_PO_4_, 1.0; MgSO_4_·7H_2_O, 0.3; agar, 20; beechwood xylan, 10, pH 5.9. The plates were incubated at 22°C for 5 days. All the plates were stained with 0.5% Congo red dye for 30 min and then washed using 1 M NaCl solution at room temperature. Zones of clearance were observed for the presence of xylanase activity (Kalim and Ali 2016). All the experiments were carried out on five replicates.

### 2.2 Sample Preparation for Mass Spectrometry Analysis

#### 2.2.1 Malt Extract Samples

To investigate the metabolites consumed and produced by *P. infestans* cultivated on a synthetic medium, the fungus was inoculated into 250 mL Erlenmeyer flasks that contained 100 mL of 2% malt extract (w/v) in distilled water (pH 5.9). Malt extract was used as the sole nutrient source. The experiment was conducted in triplicate, and two non‐inoculated flasks that contained malt extract medium were used as negative controls. All flasks were incubated by shaking at 150 rpm at 22°C for 2–4 weeks.

#### 2.2.2 Needle Samples

The fungal mycelia were transferred into 250 mL Erlenmeyer flasks that contained 100 mL of liquid medium composed of only water with 1% pine needles (w/v) cut into 5 mm pieces. The needles were collected from *P. sylvestris* trees (in July 2022, Liperi, Finland) and stored at −20°C to prevent terpene evaporation. The needles were then sterilised by gamma irradiation (Ionisos Baltics OÜ, Estonia). The experiments were conducted in triplicate. Two non‐inoculated flasks containing needle medium were used as a negative control. All the flasks were incubated with continuous shaking (150 rpm) at 22°C for 2–4 weeks (same conditions as the malt extract samples).

### 2.3 Methanol Extract Preparation

After the incubation period and when sufficient growth had been obtained, all the cultures were then filtrated using Whatman filter paper No. 1 to remove the needles and the mycelium. The filtrates were then centrifuged at 5000 rpm for 10 min to remove the remaining debris. The liquid–liquid extraction method was performed with methanol as an organic solvent. An equal volume of methanol was added to the centrifuged samples, which were then mixed thoroughly for 1 h. The mixture of methanol and samples was maintained for 10 min until two clear immiscible layers were formed. The upper methanol layer of the mixture was separated using a separation funnel (Sharma et al. 2016). The same extraction scheme was performed for the negative controls.

### 2.4 Mass Spectrometry Analysis

The compounds extracted with methanol were analysed (without chromatographic separation) by DI‐HRMS, using a flow injection analysis (FIA) technique. Briefly, the setup employed an HPLC system (Thermo UltiMate 3000 RLSCNano, Thermo Scientific, Germany) coupled to a 12‐T FT‐ICR mass spectrometer (Bruker solariX XR; Bruker Daltonics, Germany), equipped with an Apollo‐II ESI/APPI ion source (Bruker Daltonics, Germany). For the ESI experiments, 1 μL of the needle and malt methanol extracts were separately diluted with 8 mL of HPLC grade methanol. For the APPI experiments, 1 μL of methanol extracts was diluted with 1 mL of a mixture of HPLC grade methanol and toluene (1:1, v/v). Dry nitrogen was used as the drying (4.0 L/min, 200°C) and nebulizing gas (1.2 bar). The ion flight time was set at 0.7 s and the ion accumulation time was 80 ms. The mass spectra were internally calibrated during data acquisition by the signals of palmitic acid (*m/z* 255.232954; in ESI) or a toluene background ion (*m/z* 199.11174 for C_14_H_14_O_1_; in APPI). For each spectrum, 200 scans were co‐added within *m/z* 100–1000, with the time‐domain transient size of 8 MWord, providing an approximate 870,000 FWHM resolving power at *m/z* 400.

The FIA analysis was performed as follows: The diluted samples were loaded onto the autosampler, and the injection system was programmed to inject 20 μL of the sample into a 20 μL loop at a flow rate of 20 μL/min, using methanol as an eluant. The data were acquired over a 3 min period following each injection. The number of technical replicates was three, and each sample was measured three times.

The instruments were controlled, and the data were acquired using Chromeleon 6.8 (HPLC) (Thermo Fisher Scientific, Waltham, Massachusetts, USA), ftmsControl 2.1 (FT‐ICR) (Bruker, Billerica, Massachusetts, USA) and Compass HyStar 2.2 software (Bruker, Billerica, Massachusetts, USA). The data post‐processing and further analysis were accomplished using DataAnalysis 5.1 and Metaboscape 2024 software (Bruker, Billerica, Massachusetts, USA). The latter software is designed for metabolomic analysis and automatically internally recalibrates (with respect to selected analyte ions), scales and normalises the acquired data, extracts the molecular features using predefined parameters (using a T‐Rex 2D algorithm) and finally generates the most likely elemental formulae for the compounds (SmartFormula algorithm). Furthermore, it implements the CompoundCrawler database search engine for putative compound annotations using public databases, such as PubChem and ChemSpider, or the user's own internal compound libraries. A recursive feature extraction was set to filter out those features that appeared in less than six out of nine replicate measurements (Metaboscape parameter). The mass tolerance was 1 ppm, and the signal‐to‐noise (S/N) threshold was 5. The formulaic candidates were searched with the following constraints: ^12^C1–100 ^1^H0–300 ^16^O0–30 ^14^N0–2 ^32^S0–1; double bond equivalent (DBE) ≤ 50; H/C ratio ≤ 3; electron configuration: even (ESI), even/odd (APPI); mSigma ≤ 1000.

### 2.5 Statistical Analysis

One‐way ANOVA was performed using IBM SPSS Statistics (version 29.0.2.0, IBM Corp., Armonk, NY, USA) to assess the impact of different substrates on fungal growth rates and to compare the variations in extracellular enzyme secretions. Post hoc Tukey's HSD tests were conducted when the ANOVA results were significant to identify the most effective media and enzymes.

Mass spectrometry data were reported as absolute intensities. To enhance comparability across samples, including those with common and rare metabolites, the data were normalised to minimise the influence of measurement fluctuations (Sun and Xia 2024). In addition, a two‐sample *t*‐test was applied to the normalised data (Xi et al. 2014). Specifically, a two‐tailed type III *t*‐test was used to evaluate each metabolite individually. This test compared the mean values of the treatment group ( *P. infestans* samples) with the control group, which consisted only of the medium. The statistical significance of the differences in mean intensity values was determined using a *p*‐value threshold of 0.05. Furthermore, a ratio of the control mean to the sample mean (CTR/SA) was calculated to quantify and compare the observed effects between treatments.

The figures summarising the DI‐HRMS results were generated using OriginPro 2024b (OriginLab Corporation, Northampton, Massachusetts, USA).

### 2.6 Genomic Study and Pathway Reconstruction

To investigate the metabolism of the compounds detected during the mass spectrometry analysis, a pathway reconstruction was performed using the *P. infestans* genome (NCBI databank, accession JAFEVB000000000). The protein fasta generated by AUGUSTUS annotation was uploaded into the KofamKOALA webserver (https://www.kegg.jp/blastkoala/) for KEGG Orthology annotation (Aramaki et al. 2020). The COMPANION webserver (https://companion.ac.uk/) was used for protein annotation, followed by a BLAST search using BlastP on NCBI and UniProt BLAST to confirm the protein identity. The secreted proteins were determined using SecretomeP version 1.0 (http://genomics.cicbiogune.es/SECRETOOL/STP_Parser.php) (Cortázar et al. 2014). The secreted Carbohydrate‐Active Enzymes were analysed using dbCAN3 (https://bcb.unl.edu/dbCAN2/index.php) (Zheng et al. 2023). In addition, signal peptide annotation was performed using the SignalP 6.0 server (https://services.healthtech.dtu.dk/services/SignalP‐6.0/) (Teufel et al. 2022). The protein structure modelling was performed using SWISS‐MODEL (https://swissmodel.expasy.org/) (Bienert et al. 2017).

## 3 Results

### 3.1 Cultural Characteristics and Extracellular Enzyme Screening

**FIGURE 1:**
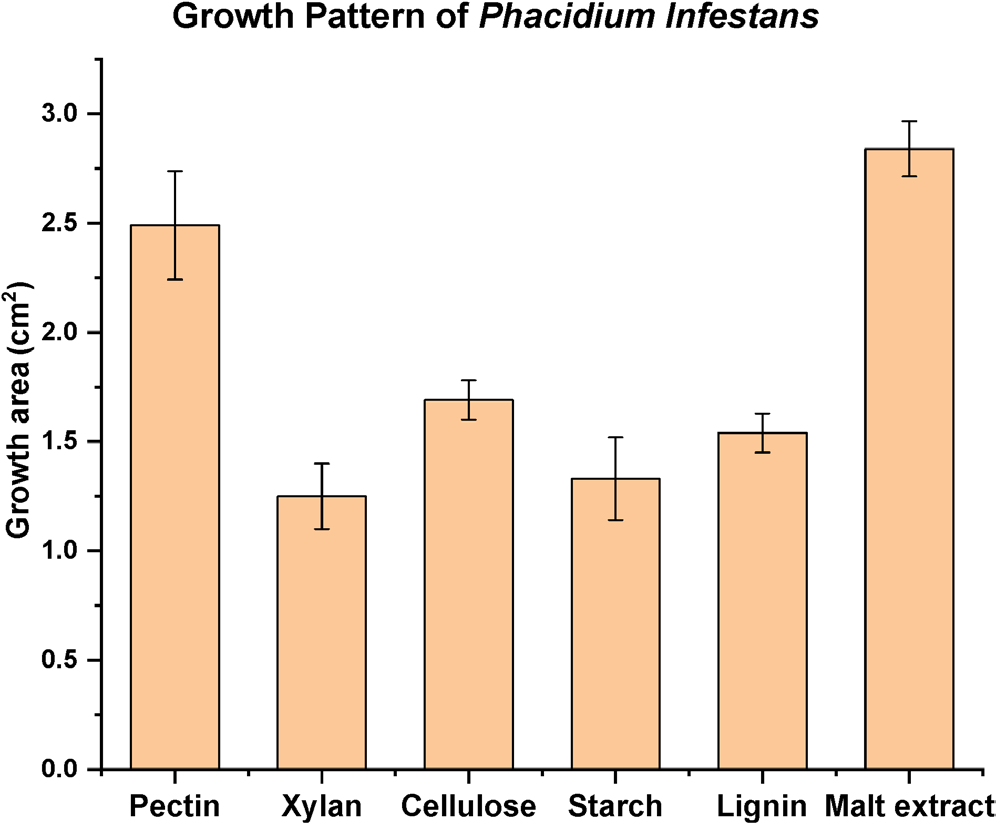
Growth pattern of *P. infestans* on agar plates that contained 1% pectin, xylan, cellulose, starch, lignin, or malt extract. The growth shown is the area of mycelial growth recorded on day 12 of cultivation.

The cultivation of the *P. infestans* DSM 5139 fungus on different media with different carbon sources indicated its ability to metabolise diverse substrates (Figure 1). The most extensive growth was recorded on malt extract, followed by pectin. The one‐way ANOVA was conducted to compare the effect of different substrates (pectin, xylan, cellulose, starch, lignin and malt extract) on fungal growth rates. The results indicated statistically significant differences between the tested substrates (*F* (5, 24) = 10.823, *p =* 1.56 × 10^−5^). Thus, the choice of substrate had a substantial impact on fungal growth. Post hoc comparisons using Tukey's HSD test revealed that malt extract promoted significantly higher growth (*p* < 0.05) compared to lignin, cellulose and xylan. Growth on pectin was also significantly higher (*p* < 0.05) than growth on xylan, cellulose and lignin. However, Tukey's HSD test showed that the difference in the fungal growth rate between the malt extract and pectin was non‐significant (*p* = 0.89). The differences in the growth rate among xylan, lignin and cellulose were also non‐significant (*p* > 0.05).

In the enzyme activity tests on agar plates, extracellular pectinase, cellulase and xylanase activities were detected (Table 1; see additional file 1, Figure S1). Based on the size of the clearance zone, the fungus strongly secreted cellulase and pectinase enzymes, whereas xylanase secretion was weaker. The analysis of variance (one‐way ANOVA) revealed a statistically significant difference between the evaluated enzyme productions (pectinase, cellulase and xylanase) (*F* (2, 12) = 77.97, *p* = 1.33 × 10^−7^), with *p* values much smaller than the conventional threshold of 0.05. Tukey's HSD test indicated that the difference in enzyme production (based on the clearance zones) was significant between all the tested enzymes (*p* < 0.05). Cellulase activity represented the most active enzyme, followed by pectinase and then xylanase. Although the fungus grew on starch, the screening for extracellular amylase yielded a negative result, which would suggest that this enzyme was potentially absent under the tested conditions (Table 1). The fungus did not grow on control plates without any added carbon source, which would indicate that it grows by utilising fibres, malt extract or starch, but not agar.

**TABLE 1:** Extracellular enzyme screening of the strain DSM 5139.

	Pectinase	Xylanase	Cellulase^a^	Amylase
Average of the clearance zone (radius, mm)^b^	14.3	8.4	16.5	—
Standard deviation	0.57	0.42	1.7	—

^a^ Activity on CMC.

^b^ Average of the clearance zone was calculated from five replicates.

### 3.2 Mass Spectrometry Analyses

The analysis of malt extract samples resulted in 340 spectral features (secondary metabolites) detected by DI‐ESI‐HRMS, from which 11 compounds were further annotated (Additional file 2; Table S1). Furthermore, DI‐APPI‐HRMS detected 1427 spectral features in the malt extract samples, and 10 compounds were identified (Additional file 2; Table S2). In total, 21 metabolites were fully annotated from the malt extract samples using both techniques.

The analysis of the needle extracts by DI‐ESI‐HRMS resulted in more than 580 spectral features; among those, 70 features were further annotated (Table S3). In contrast, about 360 spectral features (complementary to that in the ESI data) were detected with the DI‐APPI‐HRMS, out of which 42 were further annotated (Additional file 2; Table S4). Thus, a total of 112 unique metabolites were putatively identified and annotated using both techniques. The growth of *P. infestans* on the needle medium appeared as white mycelium around the needles, indicating its ability to use the needles as a sole carbon source (Additional file 1; Figure S2).

A clear difference was registered in the features identified in the studied samples (needles compared to the malt extract). The presence of resin acids was exclusively detected in the needle extracts (with and without *P. infestans* ). Analysis of malt extract samples showed that most of the annotated compounds were carbohydrates and phenolic derivatives.

### 3.3 Malt Extract Compounds Analysis

The analysis of the Malt extract samples by DI‐ESI/APPI‐HRMS served as a positive control of the growth of *P. infestans* on a synthetic medium. Most of the detected compounds were principally carbohydrates, such as maltose, D‐hexose and disaccharide (Tables S1 and S2). The medium seems quite rich in carbohydrates and malt derivatives, which contribute to effective fungal cultivation.

A statistically significant reduction of raffinose hydrate (*t*‐test significant, *p =* 1.59 × 10^−7^) with a ratio CTR/SA equal to 37.4, and glucoheptonic acid (*p =* 4.11 × 10^−8^) with a CTR/SA of 20.8 was registered in *P. infestans* samples cultivated in malt extract compared to the malt extract controls.

*P. infestans* entirely metabolised the D‐erythro‐L‐galacto‐nonulose, disaccharide, rhamnosyl‐arabinose, glucose and galactose available in the malt extract medium. The difference in the means of the two treatment groups ( *P. infestans* samples cultivated on malt extract medium and the controls) was statistically significant for all the above‐mentioned compounds (*t*‐test significant, *p* < 0.001).

### 3.4 Reconstruction of the Phenylpropanoid Pathway in *P. infestans*

**FIGURE 2:**
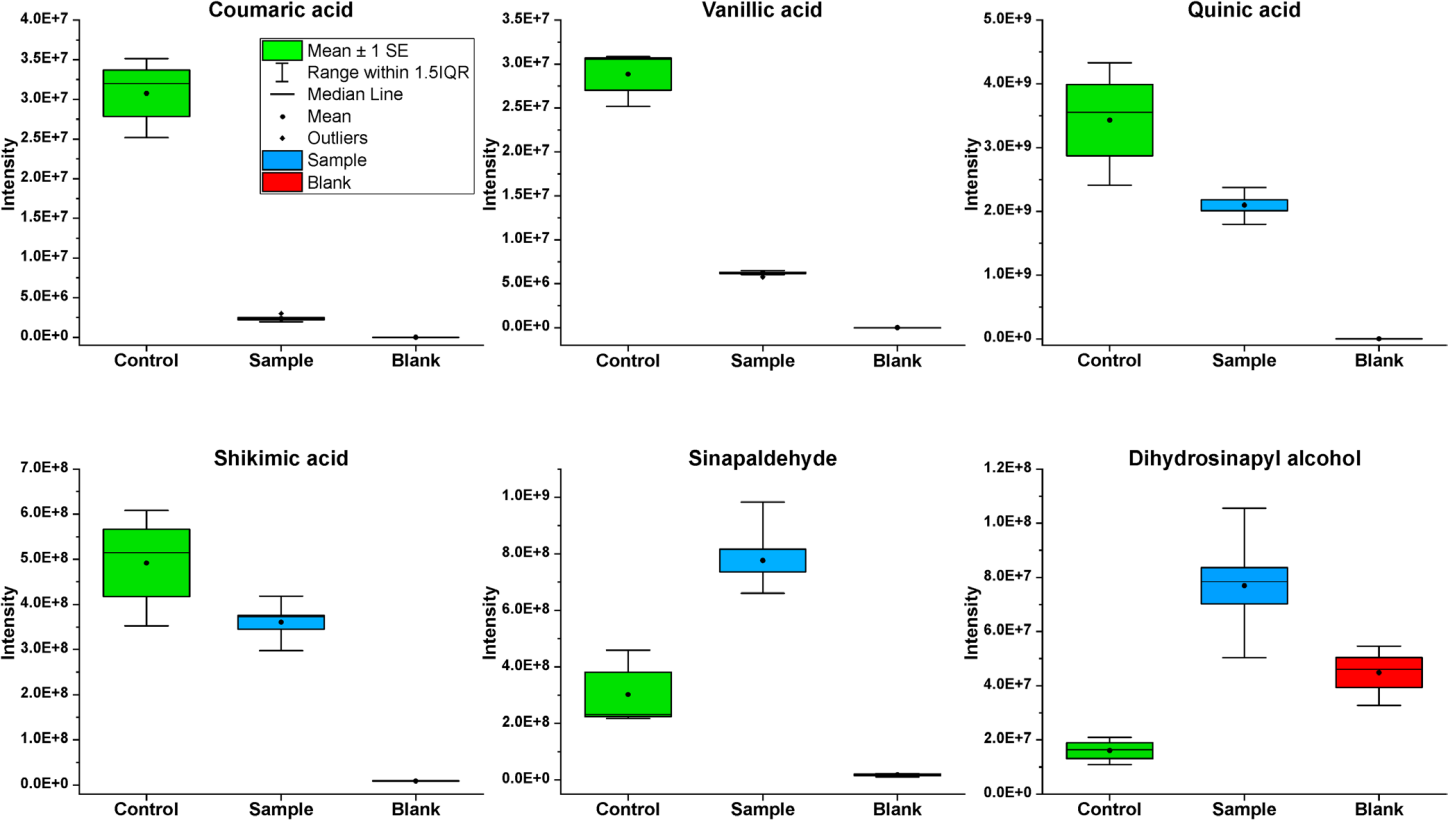
Relative abundances of coumaric acid (*p* = 0.009), vanillic acid (*p* = 0.006), quinic acid (*p* = 0.14), shikimic acid (*p* = 0.22), sinapaldehyde (*p* = 0.01), and dihydrosinapyl alcohol (*p* < 0.001) in the needle samples generated by DI(FIA)‐ESI‐HRMS. Green is the medium control (water and needles), blue indicates the samples after *P. infestans* growth, and red is the solvent control (methanol). The *p* values for each compound represent the significance of the difference between the means of the sample (with *P. infestans* ) and the control (containing only needles), as determined by a two‐sample *t*‐test.

The DI‐HRMS analysis of the needle samples showed that some of the compounds were almost completely metabolised by *P. infestans* . For instance, the relative abundance of coumaric acid and vanillic acid in the medium (water and needles) significantly decreased after fungus growth in comparison to the control (needles medium without fungus) (*p* < 0.05). This result strongly indicates the utilisation of these acids by *P. infestans* (Figure 2). Numerous compounds that were detected behaved in the same way.

Genomic annotation using the COMPANION webserver identified the protein Phain‐OT5‐proseq10905 as 4‐Coumarate: CoA ligase with 97.11% shared identity (ID) with TVY40710.1 from *L. subtilissima*. This protein catalyses the ligation of CoA to hydroxycinnamic acids (HCAs), a branch point that directs metabolites to the flavonoid or monolignol pathway. KEGG annotation identified the presence of four entries associated with the biosynthesis of various plant secondary metabolites. The analysis showed the presence of the S‐adenosylmethionine synthetase enzyme [EC:2.5.1.6], which catalyses the conversion of methionine to S‐adenosyl‐L‐methionine as part of the mugineic acid biosynthesis pathway. The pathway reconstruction analysis indicated the presence of one enzyme implicated in the coumarin biosynthesis pathway attributed to beta‐glucosidase [EC:3.2.1.21]. This enzyme acts as beta‐D‐glucosyl‐2‐coumarinate glucohydrolase and catalyses the conversion of cis‐beta‐D‐glucosyl‐2‐hydroxycinnamate to cis‐2‐hydroxycinnamate and D‐glucose. KEGG annotation also showed the presence of other enzymes implicated in coumaric acid metabolism (Additional file 1; Figure S3), such as phenacrylate decarboxylase [EC:4.1.1.102], an enzyme commonly found in fungi that catalyses the decarboxylation of phenacrylic acids present in plant cell walls. The action of the enzyme on 4‐coumarate generates 4‐vinylphenol (4‐hydroxystyrene) (Figure S3). The same enzyme can also catalyse the decarboxylation of caffeate to produce 3,4‐dihydroxystyrene (3,4‐divinylphenol) and carbon dioxide (CO_2_).

In the DI‐APPI‐HRMS analysis of the needle extracts, 5‐hydroxyferulic acid showed a CTR/SA ratio of 3.92. Similar results were observed in the malt extract samples; 5‐hydroxyferulic acid had a CTR/SA ratio of 4.96. The statistical analysis indicated a significant difference in the means of 5‐hydroxyferulic acid between the treatment groups (samples with fungus and the controls): *p* = 0.04 for the needle extracts and *p* = 0.0001 for the malt extract samples. In addition, ferulic acid was identified by DI‐APPI‐HRMS in the malt extract sample with a CTR/SA ratio of 4.71 (*t*‐test significant between the sample and the controls, *p* < 0.001) (Table S2). These results suggest the utilisation of both 5‐hydroxyferulic and ferulic acids by *P. infestans*.

DI‐ESI‐HRMS analysis of the needle extracts showed a decrease in quinic and shikimic acids in the methanol extract of pine needles after the growth of *P. infestans* (Figure 2). However, the *t*‐test results for these two compounds were statistically non‐significant. In contrast, 3‐dehydroshikimic acid showed a significant increase in the needle samples post‐ *P. infestans* growth, with a CTR/SA ratio of 0.25 (*p* = 3.17 × 10^−5^). 3‐dehydroshikimic acid may result from the oxidation of shikimic acid and can undergo further enzymatic transformations to produce quinic acid.

Genome annotation by COMPANION server indicated two entries associated with shikimic and quinic acid metabolism. Of those proteins, one was identified as quinate dehydrogenase (Phain‐OT5‐proseq8301) and shared 95.76% ID with TVY37419.1 from *L. subtilissima*. The protein Phain‐OT5‐proseq8305 was identified as quinate repressor protein (97.76% ID with TVY37421.1 from *L. subtilissima*). These annotated enzymes seem to play a role in both shikimate and quinate metabolism.

The KEGG annotation indicated the presence of the enzyme shikimate dehydrogenase [EC:1.1.1.25] in *P. infestans* . This enzyme acts as an oxidoreductase on the hydroxyl group of donors with NAD+ or NADP+ as acceptors. In this case, shikimate was converted to 3‐dehydroshikimate, thereby reducing NADP+ to NADPH and releasing protons.

The Blast of the protein Phain‐OT5‐proseq3824 on UniProt indicated a shared 98.2% ID with the strain *L. subtilissima* (gene LSUB1_G000854). The protein was identified as AROM polypeptide. This enzyme catalyses five consecutive enzymatic reactions in prechorismate polyaromatic amino acid biosynthesis. The enzyme has one cofactor and binds two Zn^2+^ ions per subunit and acts on both shikimate and quinate.

Furthermore, DI‐ESI‐HRMS analysis of the needle samples showed the formation of a greater amount (*p* < 0.05) of sinapaldehyde and dihydrosinapyl alcohol in *P. infestans* needle samples (Figure 2, Additional file 2; Table S3). Lignin is also derived from the phenylpropanoid pathway (see Figure S3). KEGG annotation indicated the presence of the enzymes catalase‐peroxidase [EC:1.11.1.21] and peroxidase [EC:1.11.1.7] in *P. infestans* . The predicted pathway seems incomplete, although the detected sinapaldehyde and dihydrosinapyl alcohol suggest the conversion of sinapaldehyde to dihydrosinapyl alcohol. In the context of lignin degradation by fungi, sinapaldehyde is an intermediate compound that can be further reduced to form simpler molecules. The presence of the enzymes responsible for the conversion and utilisation of lignin is confirmed by the cultural characterisation of *P. infestans* . Bioassays showed the capability of *P. infestans* to grow efficiently on lignin as the sole carbon source.

#### 3.4.1 Other Phenolic Compounds

Several compounds classified as polyphenols were detected in all the analysed samples. DI‐APPI‐HRMS analysis of the malt extract samples indicated that phenyl beta‐D‐glucopyranosiduronic acid (CTR/SA ratio of 1.81) significantly decreased in *P. infestans* malt extract samples compared to the control (*t*‐test significant, *p* = 6.57 × 10^−7^). Similarly, the phenolic ester acetyleugenol decreased in *P. infestans* malt extract samples with a CTR/SA ratio equal to 7.59, which would indicate substantial consumption of this compound (*p* = 3.34 × 10^−10^) (Table S2). However, a larger number of phenolic compounds were successfully annotated from the needle samples compared to the malt extract samples.

DI‐ESI‐HRMS analysis of the needle extracts (Additional file 2; Table S3) identified salicylic acid with a CTR/SA ratio of 47.24, which would indicate a considerable decrease in *P. infestans* needle samples compared to the controls without the fungus (*p* = 0.0003).

Through DI‐APPI‐HRMS of the needle extracts, two lignans (C_20_H_22_O_5_ and C_19_H_22_O_5_) were putatively identified. The amounts of all lignans significantly decreased to close to zero intensity values in *P. infestans* needle samples when compared to the controls (Table S4) (*p* < 0.001). A similar result was observed for taxiresinol, which is a type of polyphenolic compound found in plants. Taxiresinol content in *P. infestans* cultivated on needles was close to zero with a CTR/SA ratio of 298.76. Furthermore, lariciresinol, pinoresinol, allohydroxymatairesinol and demethoxypinoresinol contents significantly decreased in *P. infestans* with the needles compared to the controls (*t*‐test significant, *p* < 0.05) (Table S4). The phenolic glycoside tachioside exhibited a high CTR/SA ratio of 50.31, which indicates a significant decrease of this compound in *P. infestans* with the needles compared the controls (*p* = 0.02) (Table S3). The phenolic degradation pathways in fungi are clustered and connected by key classes of enzymes which include catechol dioxygenases (CDO). COMPANION annotation indicated the presence of three enzymes related to phenol degradation. One phenol acid carboxylase and two phenol hydrolases were detected. Protein annotation also indicated the presence of two catechol dioxygenase (Phain‐OT5‐proseq11138 and Phain‐OT5‐proseq 11232) and one catechol O‐methyltransferase (Phain‐OT5‐proseq6631, 97.48% ID with *L. subtilissima* TVY33985.1).

The DI‐APPI‐HRMS analysis of the needle samples indicated the presence of 8‐prenylnaringenin, which is also known as flavaprenin. This compound was significantly decreased in *P. infestans* needle samples (zero values) compared to the controls (*p* = 7.76 × 10^−5^). This prenylated flavonoid exhibits antifungal and antimicrobial properties.

## 4 Utilisation of Pine Needle Carbohydrates

**FIGURE 3:**
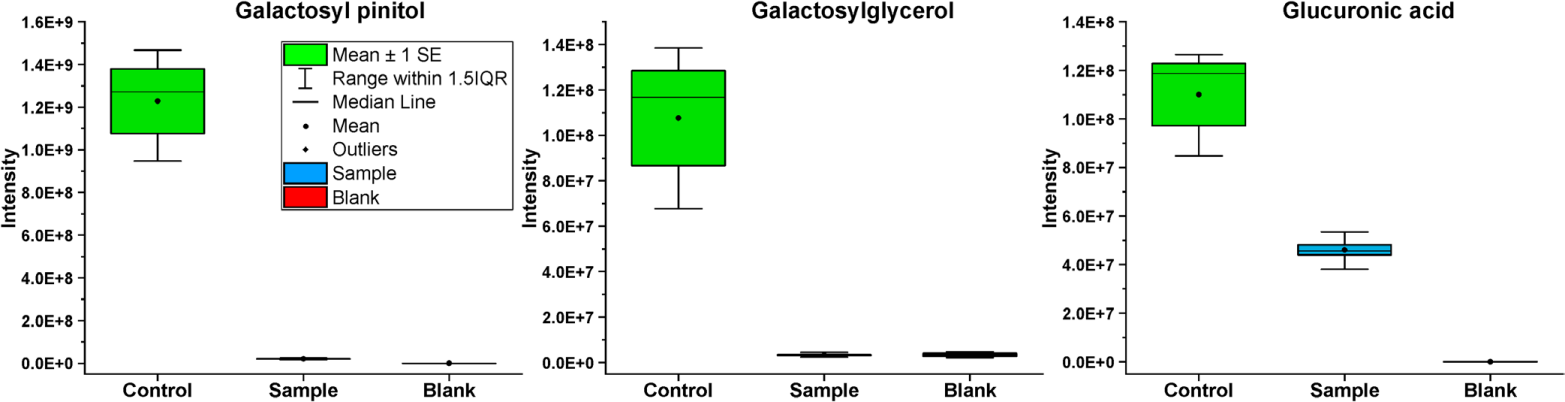
Relative abundances of galactosyl pinitol (*p* = 0.02), glactosylglycerol (*p* = 0.04), and glucuronic acid (*p* = 0.04) content of the needle samples generated by DI(FIA)‐ESI‐HRMS analysis. Green is the medium control, blue indicates the samples with *P. infestans*, and red is the solvent control (methanol). The *p* values for each compound represent the significance of the difference between the means of the sample (with *P. infestans* ) and the control (containing only needles), as determined by a two‐sample *t*‐test.

DI‐ESI‐HRMS analysis of galactosyl pinitol (Figure 3) in the needle samples showed that this compound decreased substantially after fungus growth compared to the controls (*t*‐test significant, *p* = 0.02). The ratio CTR/SA of this compound was 60.46. A similar pattern was observed for galactosyl glycerol (Figure 3), with a CTR/SA ratio of 36.60 and a significant *t*‐test value between the *P. infestans* needle samples and the controls (*p* = 0.04). Galactosyl glycerol is a glycolipid commonly found in photosynthetic tissues of plants, including pine needles.

Glucuronic acid analysis (Figure 3) showed more than a twofold decrease in *P. infestans* needle extracts compared to the controls (CTR/SA equal to 2.39, *p* = 0.04). Similarly, 4‐O‐methyl‐D‐glucaric acid content also decreased in the needle samples with *P. infestans* (CTR/SA equal to 2.45, *p* = 0.05). These results suggest that *P. infestans* appears to have utilised the hemicellulose in the needles, which typically contain D‐glucuronic acid and 4‐O‐methyl‐D‐glucuronic acid as the main components.

Analysis of sugars and their derivatives in the needle samples showed that D‐hexose, D‐glucose, methyl D‐glucoside, D‐xylose, octose, cellobiose and an unidentified disaccharide decreased in the *P. infestans* needle samples (CTR/SA ratios starting from 5.6; *p‐*values indicated on Table S3). Potential use and degradation were also observed for 5‐dehydro‐4‐deoxy‐D‐glucaric acid in the *P. infestans* needle samples. Glucoheptonic acid was found to have a CTR/SA ratio value of 46.51 in the needle samples (*p* = 0.04). Similarly, glucoheptonic acid in the malt extract indicated a CTR/SA ratio equal to 20.8 (*p* < 0.001). This strongly suggests the utilisation of this compound by *P. infestans* in the malt and needle samples.

Aside from carbohydrate utilisation, the decrease of benzoic acid in *P. infestans* needle samples compared to the controls also implies its utilisation by *P. infestans* (CTR/SA ratio of 7.46, *p* = 0.02).

### 4.1 Secreted Carbohydrate Active Enzyme Annotation

The analysis of secreted proteins in *P. infestans* was accessed by SecretomeP webtool. The predicted proteins were then submitted to dbCAN3 server for identification. In total, 421 proteins were identified as secreted and included several hypothetical proteins. Among those proteins, 128 were predicted to be carbohydrate‐active enzymes (CAZymes). A total of 117 proteins with a secretion signal were identified by at least two dbCAN out of three prediction tools. Two proteins (Phain‐OT5‐proseq10047 and Phain‐OT5‐proseq 1538) were identified as Glucoside Hydrolase family 12 (GH12) enzymes. Blastp identified these proteins as xyloglucan‐specific endo‐beta‐1,4‐glucanase and putative xyloglucanendohydrolase A (Additional file 2; Table S5). The xyloglucan‐specific endo‐beta‐1,4‐glucanase enzyme acts specifically on xyloglucan, which is a major hemicellulosic component in plant cell walls. Moreover, the protein Phain‐OT5‐proseq6582 was identified as GH74 by all dbCAN prediction tools. Uniprot confirmed the identity of this protein as Xyloglucanase with a 95% shared ID with the same protein in *L. subtilissima*. In total, 78 of the 128 predicted secreted CAZymes belonged to GH families.

Cellulase screening of *P. infestans* indicated that this enzyme exhibited the greatest level of activity detected during the biological tests. Cellulose is one of the major polysaccharides in the cell wall of most plants. Four GH5 enzymes were detected in *P. infestans* ; this family includes endoglucanase enzymes. Blastp identified Phain‐OT5‐proseq11143 as putative endo‐beta‐1,4‐glucanase B with a 94.61% shared ID with TVY20491.1 from *L. arida* . Phain‐OT5‐proseq2620 was identified as glucan 1,3‐beta‐glucosidase with a 97.82% shared ID with TVY39426.1 from *L. subtilissima*. Phain‐OT5‐proseq4458 is a putative endo‐beta‐1,4‐glucanase B, being 93.62% similar to *Lachnellula hyaline* protein XP_031000903.1. Phain‐OT5‐proseq8256 is a putative glucan endo‐1,6‐beta‐glucosidase, with a 90.97% shared ID with *L. subtilissima* TVY37118.1. All the predicted secreted proteins that specifically targeted the plant cell wall are summarised in Table S5. Eleven GH28 enzymes were identified as secreted proteins. These structurally‐related enzymes hydrolyze glycosidic bonds in pectin.

Four Carbohydrate Binding Modules (CBMs) were identified by at least two of the dbCAN3 tools. CAZypedia was used to identify these modules. CBM24 (80 residues) was found with GH71, which has an α‐1,3‐glucan degradation function. CBM42 is a module of approximately 160 residues, found mostly at the C‐terminus of GH54 catalytic domains and binds to the arabinofuranose present in arabinoxylan. The CBM43 identified is a module of 90–100 residues found at the C‐terminus of GH72. In some instances, this module carries a C‐terminal membrane anchor and has a β‐1,3‐glucan binding function. CMB20 (92 residues) was also identified by dbCAN3 as a starch‐binding domain linked to GH13.

## 5 Decomposition of Cuticular Waxes in the Needles

The cuticular waxes in the needles contain fatty alcohols and esters. Accordingly, KEGG annotation showed the ability of *P. infestans* to decompose fatty acids (Additional file 1; Figure S4). It appears that the fungus can metabolise the alcohol to aldehyde and after that to fatty acids and finally end up with alpha hydroxy fatty acid (Figure S4). COMPANION annotation indicated 49 entries associated with lipase functions. Among those, 13 GDSL‐like lipases were identified. Those enzymes catalyse the hydrolysis of ester bonds in lipids, leading to the release of fatty acids and glycerol. Six extracellular patatin‐like phospholipases that could potentially act as virulence factors were detected in *P. infestans*. Three phospholipase D‐like domains containing proteins and four lipases were identified, including one secreted lipase. The results strongly suggest the ability of *P. infestans* to degrade the fatty acids in the wax of the needles.

In *P. infestans* , three cutinase‐like sequences were identified by COMPANION annotation. Cutinase enzymes are employed by pathogens to break the cuticles that cover the aerial epidermis cells of the needles. Blastp of Phain‐OT5‐proseq3982 indicated a 96.89% shared ID with the cutinase XP_031004944.1 from *L. hyalina* . The protein was predicted by SignalP‐6.0 to possess a secretion signal. The modelling of protein Phain‐OT5‐proseq3982 using SWISS‐MODEL revealed that the most structurally similar known protein was the cutinase from *Trichoderma reesei* (PDB structure 4PSC). The sequence ID between *T. reesei* and *P. infestans* proteins was 50.25% with a Global Model Quality Estimation equal to 0.66 (QMQE). In *T. reesei* cutinase, the catalytic triad involves Ser164, His229 and Asp216 (Roussel et al. 2014). In Phain‐OT5‐proseq3982, the structure model indicated the presence of the same triad of catalytic residues Ser176, His241 and Asp228 (Additional file 1; Figure S5). The *T. reesei* cutinase possesses a lid that covers its active site (Roussel et al. 2014), which is also likely to exist in Phain‐OT5‐proseq3982, although the lid was only partially modelled. The *P. infestans* protein Phain‐OT5‐proseq3982 is likely to be a secreted and functional cutinase.

The protein Phain‐OT5‐proseq4899 shares a 98.8% ID with the cutinase TVY44579.1 from *L. subtilissima*. The protein Phain‐OT5‐proseq4899 seems to lack a signal peptide. The SWISS‐MODEL search indicated a 50% sequence ID with the structure 4PSC of *T. reesei* cutinase (Additional file 1; Figure S6). The catalytic residues Ser176, His241 and Asp228 were also detected in the protein Phain‐OT5‐proseq4899. However, the modelling did not indicate the presence of the lid, although the N‐terminal length of the protein showed that it might contain a lid structure.

A third cutinase‐like sequence, Phain‐OT5‐proseq6072, was predicted to be secreted but showed only a 30.65% shared ID with the 1cex crystal structure of *Fusarium solani* cutinase (Longhi et al. 1997). The alignment of sequences indicated that the positioning of the catalytic Asp and His was not clear and that only Ser was found in a conserved matching region.

## 6 Terpenes and Resin Acids in the Needle Samples

**FIGURE 4:**
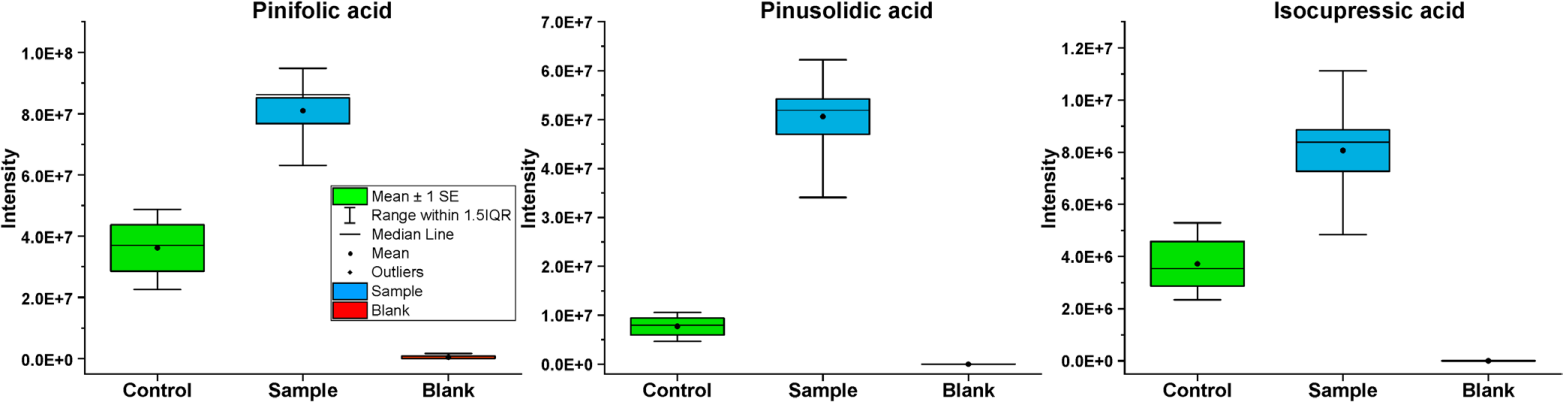
Relative abundances of some resin acid compounds identified in the needle samples: Pinifolic acid (*p* = 0.01), pinusolidic acid (*p* < 0.001), and isocupressic acid (*p* = 0.01). Green indicates the medium control, blue indicates the sample extracts from *P. infestans* cultivations, and red indicates the solvent‐control (methanol). The *p* values for each compound represent the significance of the difference between the means of the sample (with *P. infestans* ) and the control (containing only needles), as determined by a two‐sample *t*‐test.

KEGG annotation of *P. infestans* genome revealed only one entry related to pinene, assigned to hydroxymethylglutaryl‐CoA lyase [EC:4.1.3.4] and one entry for limonene degradation corresponding to aldehyde dehydrogenase (NAD+) [EC:1.2.1.3]. Both DI‐APPI‐HRMS and DI‐ESI‐HRMS analyses of the needle samples showed the presence of various resin acids, such as dehydroabietic acid, abietic acid, pinifolic acid, dehydropinifolic acid, isocupressic acid, pinusolidic acid, citronellic acid and resin acid derivatives, among others. The resin acids appear in greater concentrations (statistically significant) in the *P. infestans* with the needles compared to controls (needles without *P. infestans* ). These compounds exhibited CTR/SA ratios ranging from 0.1 to 0.5 (Figure 4 and Table S3).

The DI‐APPI‐HRMS analysis of the needle samples indicated the presence of a feature annotated as sesquiterpene and derivatives (C_15_H_20_) with a CTR/SA ratio value of 0.14, indicating that the fungus was releasing them from the needles. Sesquiterpenes and their derivatives are important constituents of pine needle essential oils (Additional file 1; Tables S3 and S4).

## 7 Genomic Identification of Virulence Factors and Drug Resistance Mechanisms

Six proteins annotated as extracellular Egh16‐like virulence factors were identified in the *P. infestans* genome. Egh16 factors play key roles in the early infection stages of pathogenic fungi. The COMPANION server identified one hypervirulence‐associated TUDOR domain‐containing protein, 14 putative mycotoxin biosynthesis proteins (UstYa), and one Zeta toxin/AAA domain‐containing protein.

*P. infestans* genome was investigated for antibacterial and antifungal resistance. The mechanisms of antifungal drug resistance include the reduction of drug–target interactions and the reduction in intracellular drug levels using drug pumps. Three Multidrug/Oligosaccharidyl‐lipid/Polysaccharide (MOP) flippase superfamily proteins and three pleiotropic drug resistance proteins (Phain‐OT5‐proseq5913, Phain‐OT5‐proseq8177, Phain‐OT5‐proseq7851) were identified. In addition, 38 proteins were predicted by COMPANION server as putative fungal trichothecene efflux pumps (TRI12) and 12 proteins as Drug/Metabolite Transporter (DMT) Superfamily members.

COMPANION analysis indicated the presence of two proteins (Phain‐OT5‐proseq999 and Phain‐OT5‐proseq3542) as the putative Acyclic terpene utilisation family protein AtuA. These proteins might be involved in the uptake and transport of acyclic terpenes and terpene detoxification.

## 8 Discussion

*P. infestans* is known to survive in harsh environments. This cold‐adapted fungus also faces other challenges. *P. infestans* grows on pine needles and tolerates their high terpene content (Manninen et al. 2002). In this study, the capability of the fungus to grow on pine needles and degrade them was investigated by DI‐HRMS analysis and supplemented with genomic data. In addition, the fungus was cultivated on malt extract medium and further analysed by DI‐HRMS.

The DI‐HRMS analysis of *P. infestans* cultivated on malt extract revealed a high consumption of carbohydrates compared to the malt extract controls. Most of the identified compounds are sugars, which emphasise the importance of using malt extract medium to promote fungal growth.

In the needle extracts, numerous resin acids and their derivatives were detected. These compounds were exclusively present in needle samples (not detected in the malt extract samples). Dehydroabietic acid, abietic acid, pinifolic acid, pinusolidic acid, isocupressic acid and other resin acids were significantly higher (*p* < 0.05) in *P. infestans* samples cultivated on the needles compared to the controls. Pine needle epithelial cells are known to synthesise, store and secrete resin, which is comprised of a complex mixture of compounds that include terpenes and resin acids (Bouwmeester 2019). The pinifolic acid and the abietic acid identified in this study are the primary components of the resin found in *P. sylvestris* needles (Andersson et al. 1990). These compounds belong to the abietane diterpene family and, like other resin acids, are crucial for the defence mechanisms of pine trees (Hamberger et al. 2011).

Since the resin acids were detected only in the needle extracts and not in the malt extracts, their increased levels after *P. infestans* growth on the needles suggest their release during the fungal degradation of the needle cell wall. The genomic identification of several multidrug efflux pumps suggests the ability of *P. infestans* to transport toxic components, such as the identified resin acids, out of the cell. Therefore, the fungus might be able to efficiently conserve its homeostasis and integrity in this highly chemical and toxic environment (Tsuchiya et al. 2016).

To address the hypothesis of resin acid release, the potential mechanisms of host penetration by the fungus were examined. The cuticular wax on the needles is recognised for its protective function (Agrios 2005). It acts as the first line of defence against biotic and abiotic stresses (Arya et al. 2021). Thus, the strategies employed by the fungus to break down the waxes were explored. The cuticular wax of pine needles is primarily composed of long‐chain fatty acids (21%) (Wen et al. 2006). Genomic annotation of *P. infestans* indicated the presence of several extracellular lipases including GDSL‐like lipases that may be able to degrade those fatty acids. Previous research on the *Acidovorax citrulli* mutant defective in lip1, a gene that encodes a GDSL‐lipolytic enzyme, highlighted the contribution of this lipolytic enzyme to the virulence of plant pathogens that belong to the *Acidovorax* genus (Rosenberg et al. 2022). In *F. graminearum*, the causal agent of *Fusarium* head blight (FHB) and a destructive pathogen of cereals, previous studies have indicated that a reduced extracellular lipolytic activity in culture could lead to reduced virulence (Voigt et al. 2005). In addition, aerial plant organs are often protected by a cuticle that is composed of cutin, a polyester composed of hydroxy and hydroxyepoxy fatty acids (Dutta et al. 2009). Cutinases can catalyse the breakdown of the polyesters that form the cuticle that protects plants. These enzymes are often synthesised by phytopathogenic fungi. In this study, three putative cutinases were identified and modelled, from which two seem to be functional. One cutinase was secreted, and the other may be intracellular. Previous studies on a cutinase without a signal peptide expressed in *E. coli* showed that cutinase activity was detected in the culture medium. The cells that express the cutinase without a signal peptide presented an irregular morphology and increased membrane permeability, potentially leading to ‘cell leakage’ anf thus explaining the extracellular release of the non‐secreted cutinase (Su et al. 2013). This would indicate that both intracellular and extracellular cutinases play a role in the breakdown of cutin. Cutinase enzymes are crucial for infection in certain host–pathogen interactions (Dickman et al. 1989). *P. infestans* may utilise its cutinase to effectively penetrate the cuticular wax layer of pine needles.

Phytopathogenic fungi are known to secrete cell wall degrading enzymes to break down complex polysaccharides in the plant cell wall. These enzymes play significant roles in host invasion and virulence (Rafiei et al. 2021). To penetrate and break down plant barriers, most phytopathogenic fungi and oomycetes have developed an arsenal of enzymes that include pectinases, polygalacturonases, glucanases, cellulases and xyloglucanases (Nühse 2012). In this study, the fungal growth of *P. infestans* was initially evaluated on various substrates, such as lignin, cellulose, pectin and xylan, to determine its ability to utilise them as a unique carbon source. The results revealed that aside from its growth on the tested substrates, the fungus secreted extracellular cellulase, pectinase and xylanase. Most of these enzymes are employed to destroy the plant cell wall components, which include cellulose, cross‐linking glycans, hemicellulose and pectins (Vetchinkina et al. 2022). Therefore, the initial enzyme bioassays confirmed the capacity of *P. infestans* to degrade the plant cell wall polymers. The genome annotation of *P. infestans* showed that the GH28 family contained the largest number of secreted proteins (Table S5). Eleven secreted GH28 family proteins were detected; among those, six secreted polygalacturonases. Pectin degradation is considered to strongly contribute to fungal virulence, but it also induces defence gene expression in the host plant (Herbert et al. 2003). In plant pathogenic fungi, polygalacturonases are employed in fungal penetration, tissue collapse and nutrient acquisition by degrading adhesive pectic substances in the middle lamella and cell wall (Nakamura and Iwai 2019). The presence of several GH28 pectinases has been demonstrated in saprotrophs that grow on senesced leaf tissues and in plant pathogens that seek to gain access to plant intracellular nutrients. Previous studies on these proteins have suggested their lineage‐specific expansions in necrotrophic fungal pathogens (Sprockett et al. 2011). In addition, xyloglucanases specifically hydrolysing the xyloglucan are indispensable in the invasion of plants by pathogens (Jiang et al. 2023). *P. infestans* genomic annotation indicated the presence of two secreted xyloglucanases and one putative xyloglucan endohydrolase, in addition to several other cell wall degrading enzymes (Table S5).

The DI‐APPI/ESI HRMS analysis showed a significantly decreased concentration of many compounds in the *P. infestans* needle samples. The initial content of coumaric acid in the pine needles significantly decreased after *P. infestans* fungus growth. Previous studies have shown the presence of coumaric acid (among other phenolic compounds) in both water and ethanol extracts of spruce needles (Mofikoya et al. 2023). Coumaric acid is also a major precursor in the synthesis of other phenolic acids, such as caffeic, chlorogenic, rosmarinic and ferulic acids (Jiang et al. 2016; Pragasam et al. 2013). Studies on p‐coumaric acid and its conjugated forms have revealed antioxidant, antimicrobial, antitumor and anti‐inflammatory properties (Pei et al. 2016). HCAs, such as cinnamic acid, coumaric acid, sinapic acid, and others, have been reported to protect plant cells against pathogen invasion by strengthening cell walls or by acting as direct antimicrobial agents (Campos et al. 2014). P‐Coumaric acid confers protection against infection by *Xanthomonas campestris* pv. Campestris (Islam et al. 2018). It has also been found to increase chitinase activity in leaves and β‐1,3‐glucanase activity in roots, thereby enhancing the resistance of the watermelon ( *Citrullus lanatus* ) to *F. oxysporum* (Ren et al. 2016). Additionally, p‐coumaric acid has been shown to inhibit the growth of *Colletotrichum acutatum* (Roy et al. 2018). However, previous research on *Ralstonia solanacearum* has indicated that this fungus can protect itself from HCA toxicity by using low concentrations of HCA as a carbon source (Lowe et al. 2015). The reduced levels of coumaric acid on pine needles after fungus growth indicate that *P. infestans* utilises the compound in its metabolism. In addition, vanillic acid was one of the compounds that significantly decreased in *P. infestans* needle samples compared to the controls. Vanillic acid is a phenolic compound (4‐hydroxy‐3‐methoxy benzoic acid) that is produced by many plants as a secondary metabolite that exhibits antimicrobial properties (Tijerina‐Ramírez et al. 2014). Vanillic acid was previously investigated for its antifungal activity against the phytopathogenic fungus *S. rolfsii* and demonstrated a significant inhibitory effect on the growth, morphology and biochemical attributes of *S. rolfsii*, where growth was completely constrained at a concentration of 0.025% (Yousaf et al. 2023). On the other hand, it seems that many fungal strains can tolerate vanillic acid (0.5 g/L), and 50% of the strains that grow on solid media have been shown to assimilate vanillic acid (Guiraud et al. 1992). The metabolism of vanillic acid, a product of lignin degradation, has also been previously investigated in soft‐rot, brown‐rot and white‐rot fungi (Buswell et al. 1982), as well as in the wild type of white–rot fungus *Sporotrichum pulverulentum*. Vanillic acid was found to be oxidatively decarboxylated to methoxyhydroquinone (MHQ) and simultaneously reduced to vanillin and vanillyl alcohol to different degrees depending on the cultivation conditions (Ander et al. 1980).

The concentrations of shikimic acid and quinic acid decreased by up to two‐fold in the *P. infestans* extracts. These compounds are part of the same biosynthesis pathway. Based on KEGG annotation, the shikimate pathway, which involves phosphoenolpyruvate and erythrose‐4P resulting in chorismite, is complete in *P. infestans*. The shikimate pathway is responsible for the synthesis of vitamins and aromatic amino acids (Bentley and Haslam 1990). These amino acids are not only vital for the protein synthesis of the fungi but also contribute to the production of the secondary metabolites involved in the interaction with the host plant. Since the shikimate pathway is essential for the survival of plant pathogenic fungi, it serves as a potential target for fungicidal compounds. Inhibition of this pathway can disrupt the synthesis of essential aromatic compounds and lead to the death of the fungus (Kuplińska and Rząd 2021). In *Aspergillus nidulans*, the shikimate pathway and the quinic acid utilisation (QUT) have two common metabolic intermediates, 3‐dehydroquinic acid (DHQ) and dehydroshikimic acid (DHS), which are interconverted by two isoenzymes, catabolic 3‐dehydroquinase (cDHQase) and biosynthetic dehydroquinase (bDHQase). The latter is one of five consecutive enzymes associated with the pentafunctional AROM protein encoded by the complex AROM (Aromatic Amino Acid), whereas cDHQase is encoded by the single‐function *QUTE* gene, which is required for the catabolism of quinate to protocatechuate (Lamb et al. 1991). Similar biosynthetic reactions probably exist in *P. infestans* , involving the identified pentafunctional AROM protein, which is known to play a central role in the shikimate pathway.

The needles and bark of *Pinus* species are known to contain various polyphenols (Cannac et al. 2007). In this study, analysis of phenolic compounds in the needle samples indicated that *P. infestans* completely metabolised several lignans, such as taxiresinol, lariciresinol, pinoresinol, allohydroxymatairesinol and demethoxypinoresinol. Lignans occur mainly in the knots of pine trees and Scots pine (*Pinus silvestris*). Knots have been found to contain 0.4%–3% lignans (Holmbom et al. 2003). Studies on taxiresinol have indicated its potential antifungal activity on *F. solani* growth (Prieto et al. 2013) and its moderate antifungal activity against *Trichophyton longifusus* and *Microsporum canis* (Erdemoglu et al. 2004). Lariciresinol exhibits antifungal properties against several human pathogenic fungal strains (Hwang et al. 2011). Previous studies on pinoresinol have shown its antifungal activity against numerous human pathogenic fungi, including *Candida albicans* , *Trichosporon beigelii* and *Malassezia furfur*. Pinoresinol acts by damaging the fungal plasma membrane (Hwang et al. 2010). In addition, 8‐prenylnaringenin significantly decreased in *P. infestans* with the needles. This prenylated flavonoid is one of the most potent in vitro phytoestrogens known (Pohjanvirta and Nasri 2022) and has antimicrobial and antifungal properties (Mizobuchi and Sato 1984; Osorio et al. 2021).

The samples that contained *P. infestans* cultivated on the needles showed a significant decrease in the salicylic acid content compared to the controls (over 40‐fold). Salicylic acid is a key plant defence hormone that plays an important role in local and systemic responses against biotrophic pathogens (Rabe et al. 2013). Salicylic acid exhibits antifungal potential through the reduction and prevention of the growth of pathogens (Koo et al. 2020). Many fungi, including non‐pathogenic species, can degrade salicylic acid to manipulate the plant defence response (Lubbers et al. 2021). The first key enzymes implicated in its degradation were identified in *F. graminearum* as salicylate 1‐monooxygenase and catechol 1,2‐dioxygenase (Rocheleau et al. 2019). Genomic annotation of *P. infestans* has identified three enzymes related to the phenol pathway, two catechol dioxygenases and one catechol O‐methyltransferase. Previous research by Soal et al. (2022) focused on the evolution of catechol dioxygenases in *Ceratocystidaceae* species and their potential links with fungal lifestyle. The study showed that the genomes of the necrotrophic pathogens, aside from *B*. *fagacearum*, contained four different genes that encode CDOs, mildly pathogenic species only contained two to three genes and saprophytic species only contained a single gene. Catechol 1,2‐dioxygenases have been studied for their ability to cleave the benzene ring of catechol, which is the main intermediate in the degradation of aromatic compounds (Rodríguez‐Salazar et al. 2020). Catechol degradation is an important process in the context of phytopathogenic fungi as this compound is a key intermediate in the breakdown of aromatic compounds that include certain plant defence compounds. Therefore, the breakdown of catechol can influence their pathogenicity and their ability to overcome plant defences (Rodríguez‐Salazar et al. 2020).

## 9 Conclusions

Using DI‐HRMS, this research explored the growth dynamics of *P. infestans*, also known as snow mould, on pine needles as the sole carbon source. The study was complemented by cultural characterisation, enzyme screening and genome annotation. The results indicated the ability of *P. infestans* to successfully penetrate the cuticular waxes of the needles and acquire nutrients, thereby utilising needle resources, such as cell wall polymers and carbohydrates. Moreover, *P. infestans* utilised several lignans and phenols and appeared to maintain fungal homeostasis by the metabolisation (or by expelling) of large numbers of toxic compounds. Moreover, *P. infestans* reduced the relative abundance of numerous antifungal compounds of pine needles to indetectable levels, which would suggest their utilisation for its metabolism. These results contribute to our understanding of plant‐pathogen interactions and nutrient acquisition and emphasise the need for comprehensive studies to unravel the molecular and physiological responses of both the pathogen and its host in the context of such unique ecological niches.

## Author Contributions

**Chahira Zerouki:** conceptualization, investigation, funding acquisition, writing – original draft, writing – review and editing, validation, methodology, visualization, software, formal analysis, data curation. **Omolara Mofikoya:** formal analysis, writing – review and editing, software, writing – original draft. **Taskeen Badar:** writing – review and editing, formal analysis. **Marko Mäkinen:** writing – review and editing, conceptualization, visualization, validation, data curation, software. **Ossi Turunen:** writing – review and editing, supervision, resources, project administration, visualization, methodology. **Janne Jänis:** writing – review and editing, supervision, resources, software, project administration, visualization, methodology.

## Ethics Statement

This study did not involve any human subjects, animal experimentation or other ethical considerations. Therefore, no ethics approval was required for this research.

## Conflicts of Interest

The authors declare no conflicts of interest.

## Supporting information


**Figure S1.** Pectinase activity of *P. infestans* on 1% pectin minimal medium revealed by iodine staining.
**Figure S2.** *P. infestans* growth on medium containing needles as the sole carbon source. The image was taken after 14 days of cultivation.
**Figure S3.** Putative phenylpropanoid biosynthesis pathway of *P. infestans* generated by BlastKOALA. The enzymes detected in *P. infestans* are shown in green. Red indicates all the potential pathways including incomplete pathways.
**Figure S4.** Putative fatty acid decomposition pathway of *P. infestans* as generated by BlastKOALA.
**Figure S5.** Amino acid numbering of Phain‐OT5‐proseq3982, a putative cutinase. The red amino acids indicate the positions of the catalytic triad amino acids Ser176, His241 and Asp228.
**Figure S6.** Alignment of the protein sequences Phain‐OT5‐proseq3982 and Phain‐OT5‐proseq4899 from *P. infestans* with the cutinase sequence 4PSC_1, chain A from *Trichoderma reesei*.
**Table S1.** Mass spectrometry results of the annotated compounds from the MEA extracts identified by DI‐ESI‐HRMS.
**Table S2.** Mass spectrometry results of the annotated compounds from the MEA extracts identified by DI‐APPI‐HRMS.
**Table S3.** Mass spectrometry results of the annotated compounds from the needle extracts identified by DI‐ESI‐HRMS.
**Table S4.** Mass spectrometry results of the annotated compounds from the needle extracts identified by DI‐APPI‐HRMS.
**Table S5.** Secreted proteins of *P. infestans* implicated in plant cell wall degradation as annotated by dbCAN3.
**Data S1.** The predicted protein sequences used in the study.

## Data Availability

The *P. infestans* genome is publicly available from the NCBI database with the accession JAFEVB000000000. The protein sequences used in this article are included in the supplemental file of this article (Data S1). The Metabolomic data are deposited in a public data repository and are available from https://data.eu‐fticr‐ms.eu/.
