# Supplementary material for: Metabolomic and Genomic Analysis of Bioactive Compounds of Phacidium infestans Karsten DSM 5139 Cultivated on Pinus sylvestris Needles
Source: Environ Microbiol Rep. 2025 Jun 9;17(3):e70084. doi: 10.1111/1758-2229.70084 (PMC12148953; doi:10.1111/1758-2229.70084)
Supplement: Supplementary file 1 — Figure S1. Pectinase activity of P. infestans on 1% pectin minimal medium revealed by iodine staining. Figure S2. P. infestans growth on medium containing needles as the sole carbon source. The image was taken after 14 days of cultivation. Figure S3. Putative phenylpropanoid biosynthesis pathway of P. infestans generated by BlastKOALA. The enzymes detected in P. infestans are shown in green. Red indicates all the potential pathways including incomplete pathways. Figure S4. Putative fatty acid decomposition pathway of P. infestans as generated by BlastKOALA. Figure S5. Amino acid numbering of Phain‐OT5‐proseq3982, a putative cutinase. The red amino acids indicate the positions of the catalytic triad amino acids Ser176, His241 and Asp228. Figure S6. Alignment of the protein sequences Phain‐OT5‐proseq3982 and Phain‐OT5‐proseq4899 from P. infestans with the cutinase sequence 4PSC_1, chain A from Trichoderma reesei. Table S1. Mass spectrometry results of the annotated compounds from the MEA extracts identified by DI‐ESI‐HRMS. Table S2. Mass spectrometry results of the annotated compounds from the MEA extracts identified by DI‐APPI‐HRMS. Table S3. Mass spectrometry results of the annotated compounds from the needle extracts identified by DI‐ESI‐HRMS. Table S4. Mass spectrometry results of the annotated compounds from the needle extracts identified by DI‐APPI‐HRMS. Table S5. Secreted proteins of P. infestans implicated in plant cell wall degradation as annotated by dbCAN3. Data S1. The predicted protein sequences used in the study. [file EMI4-17-e70084-s001.docx]

**Metabolomic and genomic analysis of bioactive compounds of *Phacidium infestans* Karsten DSM 5139 cultivated on *Pinus sylvestris* needles**

Zerouki. C^1.2*^., Mofikoya. O^3^., Badar. T^1^., [Mäkinen](https://pubmed.ncbi.nlm.nih.gov/?term=M%C3%A4kinen+M&cauthor_id=32426612). M^3^., Turunen. O^1#^ & Jänis. J^3#^

^1^School of Forest Sciences, University of Eastern Finland. P.O. Box 111. FI-80101 Joensuu. Finland

^2^Department of Environmental and Biological Sciences, University of Eastern Finland. P.O. Box 111. FI-80101 Joensuu. Finland

^3^Department of Chemistry and Sustainable Technology, University of Eastern Finland. P.O. Box 111. FI-80101 Joensuu. Finland

*Corresponding author: [czerouki@uef.fi;](rewritten://4c3437af-e7ba-4ba9-8823-e334a2bd4b12) [chahira.zerouki@uef.fi](mailto:chahira.zerouki@uef.fi); https://orcid.org/0000-0002-3285-5818

^#^Both senior authors contributed equally to the study

**Additional file 1**


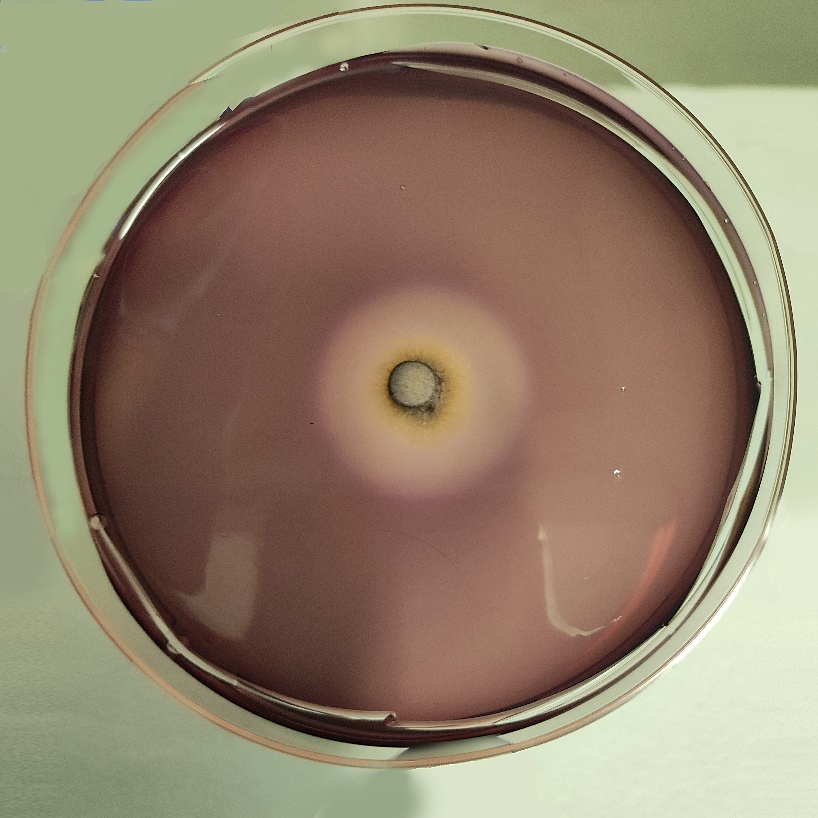


**Fig. S1.**  Pectinase activity of *Phacidium infestans* on 1% pectin minimal medium as revealed by iodine staining.


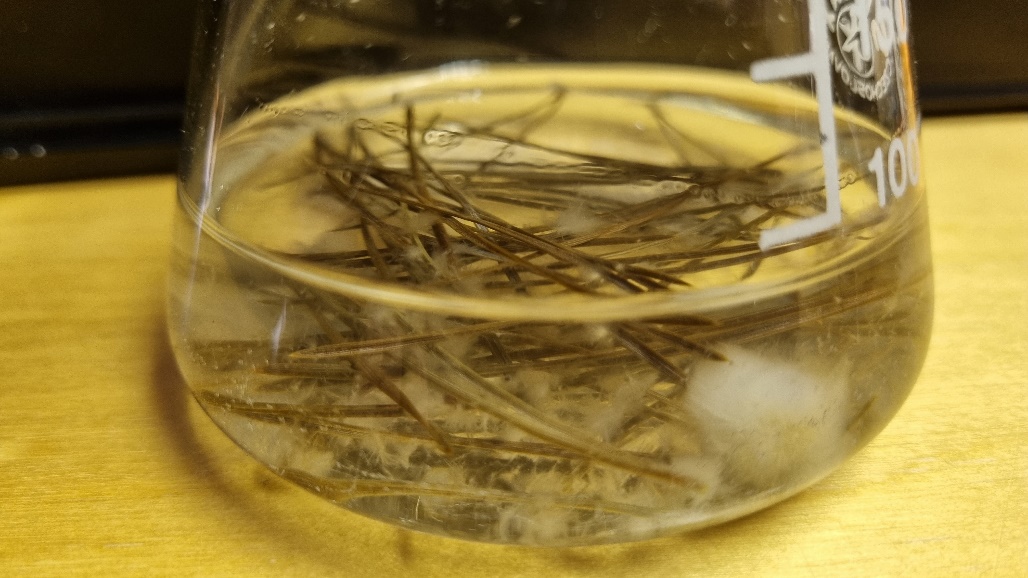


**Fig. S2.** *Phacidium infestans* growth on medium containing needles as the sole carbon source. The image was taken after 14 days of cultivation.


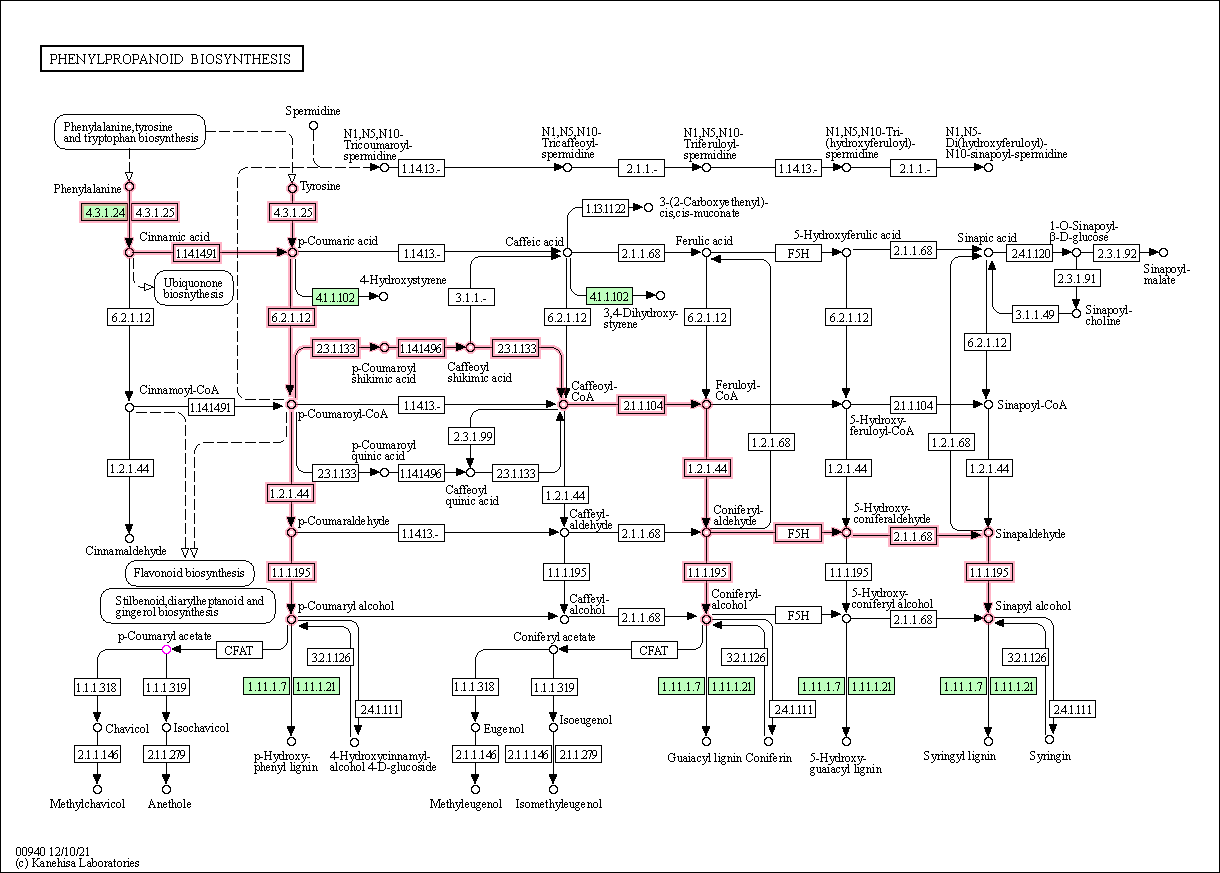


**Fig. S3.** Phenylpropanoid biosynthesis pathway of *Phacidium infestans* as generated by BlastKOALA. Enzymes detected in *P. infestans* are shown in green. Red indicates all the potential pathways including incomplete pathways.


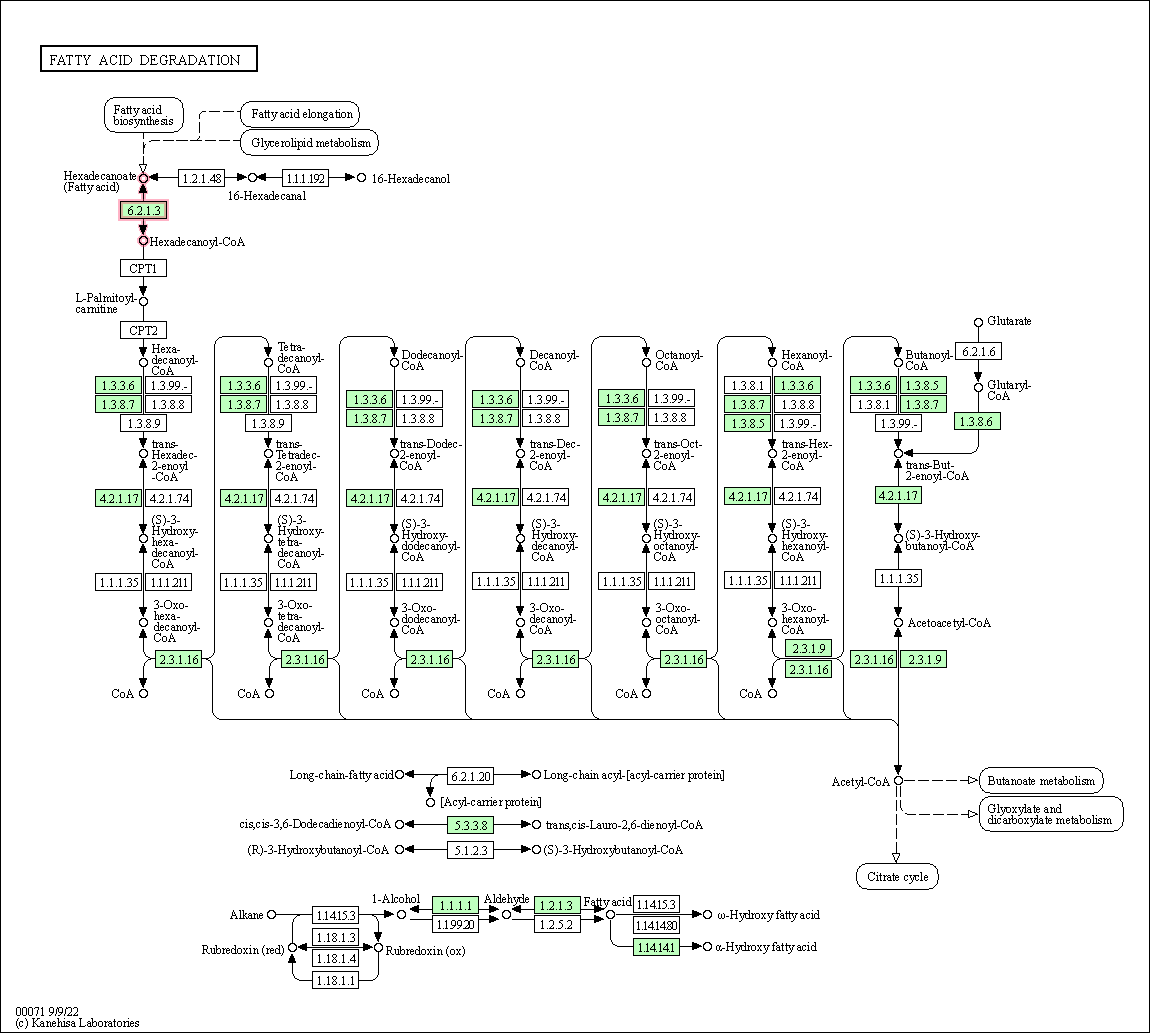


**Fig. S4**. Fatty acid decomposition pathway of *Phacidium infestans* as generated by BlastKOALA.


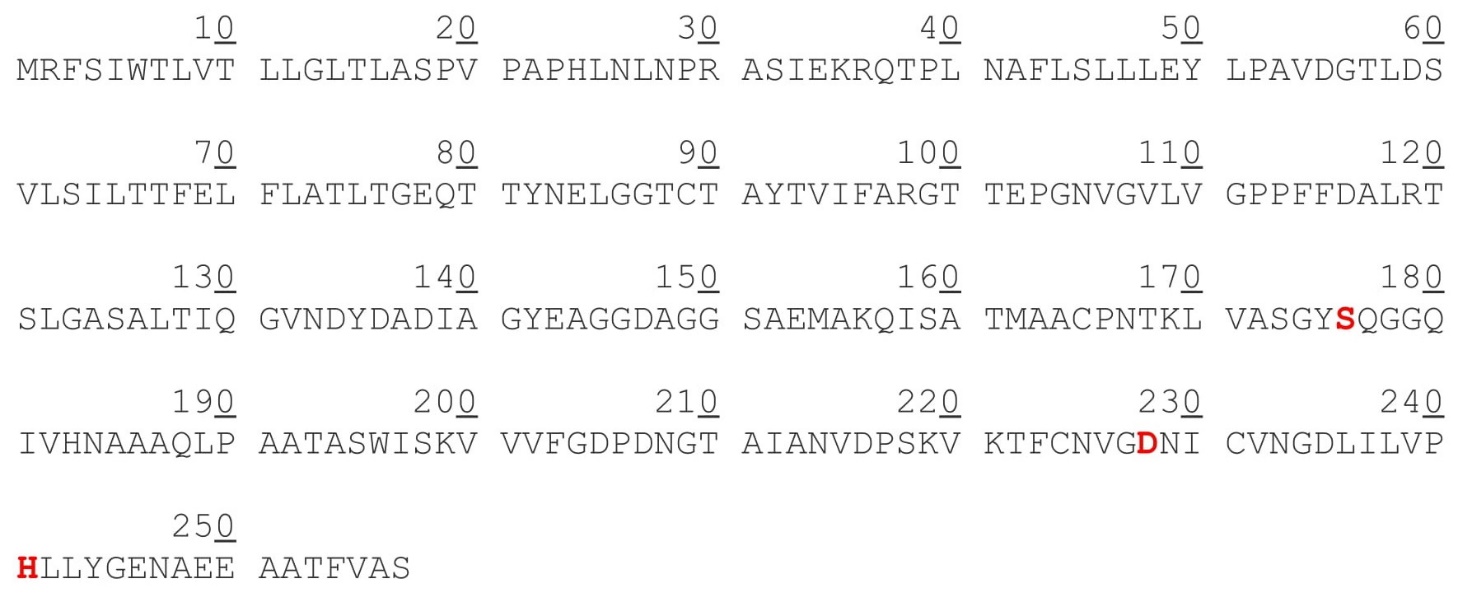
 **Fig. S5.** Amino acid numbering of Phain-OT5-proseq3982, a putative cutinase. Red amino acids indicate the positions of the catalytic triad amino acids Ser176, His241, Asp228.


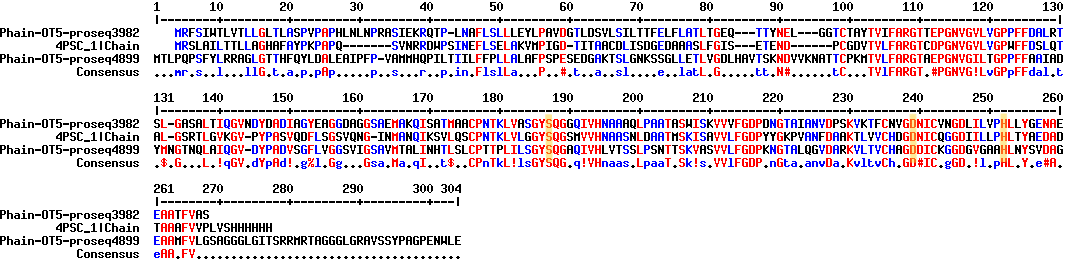


**Fig. S6.** Alignment of the protein sequences Phain-OT5-proseq3982 and Phain-OT5-proseq4899 from *Phacidium infestans* with the cutinase sequence 4PSC_1, chain A from *Trichoderma reesei.*

**Additional file 2**

| **Table S1**. Mass spectrometry results of the annotated compounds from the MEA extracts identified by DI-ESI-HRMS. | | | | | | | | | |
| --- | --- | --- | --- | --- | --- | --- | --- | --- | --- |
| Compound | Molecular  Formula | Control* (average) | Control * SD (±) | Sample* (average) | Sample* SD (±) | Blank*  (methanol average) | Blank*  SD (±)  (Methanol) | *p-*value# | Ratio  CTR/SA |
| Maltose | C_12_H_22_O_11_ | 6.93E+08 | 55799329 | 7.26E+08 | 70522236 | 8.05E+05 | 1610411 | 0.355 | 0.96 |
| levoglucosan | C_6_H_10_O_5_ | 4.21E+08 | 12880065 | 4.73E+08 | 25296299 | 3.76E+06 | 1534229 | 0.0004 | 0.89 |
| D-hexose | C_6_H_12_O_6_ | 4.76E+08 | 17825329 | 1.81E+08 | 10122495 | 9.49E+06 | 6534060 | 1.28E-09 | 2.63 |
| Maltotriose | C_18_H_32_O_16_ | 1.21E+08 | 7046523 | 1.07E+08 | 8570572 | 0 | 0 | 0.007 | 1.13 |
| Glucose, galactose | C_12_H_24_O_12_ | 5.26E+07 | 4756195 | 0 | 0 | 0 | 0 | 1.29E-06 | - |
| Rhamnosyl-arabinose | C_11_H_20_O_9_ | 4.83E+07 | 5168310 | 0 | 0 | 0 | 0 | 2.95E-06 | - |
| Raffinose hydrate | C_18_H_34_O_17_ | 3.59E+07 | 2929214 | 9.60E+05 | 1028091 | 0 | 0 | 1.59E-07 | 37.4 |
| glucoheptonic acid | C_7_H_14_O_8_ | 1.76E+07 | 1822407 | 8.47E+05 | 1182677 | 0 | 0 | 4.11E-08 | 20.8 |
| Dissacharide | C_12_H_22_O_10_ | 1.87E+07 | 1605029 | 0 | 0 | 0 | 0 | 9.88E-07 | - |
| D-erythro-L-galacto-Nonulose | C_9_H_18_O_9_ | 1.78E+07 | 3263403 | 0 | 0 | 0 | 0 | 4.20E-05 | - |
| D-Rhamninose | C_18_H_32_O_14_ | 6.42E+06 | 452997 | 7.86E+06 | 1501518 | 0 | 0 | 0.031 | 0.82 |

* Values of this table are the absolute intensity normalized and scaled using pareto scaling.

# Calculated using t-test, tails 2, type 3 between the control values and the samples.

| **Table S2.** Mass spectrometry results of the annotated compounds from the MEA extracts identified by DI-APPI-HRMS. | | | | | | | | | |
| --- | --- | --- | --- | --- | --- | --- | --- | --- | --- |
| Compound | Molecular  Formula | Control* (average) | Control * SD (±) | Sample* (average) | Sample* SD (±) | Blank*  (methanol average) | Blank*  SD (±)  (Methanol) | *p-*value# | Ratio  CTR/SA |
| 5-hydroxyferulic acid | C_10_H_10_O_5_ | 8.87E+08 | 130575882 | 6.23E+08 | 178729250 | 0 | 0 | 0.0001 | 4.96 |
| Phenyl beta-D-glucopyranosiduronic acid | C_12_H_14_O_7_ | 4.09E+08 | 82153884 | 8.25E+08 | 225647860 | 0 | 0 | 6.57E-07 | 1.81 |
| Maltol galactoside | C_12_H_16_O_8_ | 4.91E+08 | 110989587 | 7.60E+08 | 108108096 | 0 | 0 | 7.31E-06 | 4.54 |
| Maltosan | C_12_H_20_O_10_ | 5.92E+08 | 85276577 | 5,59E+08 | 210984852 | 0 | 0 | 0.57 | 2.81 |
| Acetyleugenol | C_12_H_14_O_3_ | 4.23E+08 | 50201519 | 1.90E+08 | 55679441 | 0 | 0 | 3.34E-10 | 7.59 |
| Dihydroresveratrol | C_14_H_14_O_3_ | 9.50E+07 | 1511947 | 1.26E+08 | 36220567 | 0 | 0 | 0.006 | 2.62 |
| Menadiol | C_11_H_10_O_2_ | 9.54E+07 | 12687896 | 1.08E+08 | 28103086 | 0 | 0 | 0.12 | 3.39 |
| Ferulic acid | C_10_H_10_O_4_ | 3.54E+07 | 4090178 | 2.32E+07 | 7513009 | 0 | 0 | 1.21E-05 | 4.71 |
| Maltol 6'-O-beta-D-apiofuranosyl-beta-D-glucopyranoside | C_17_H_24_O_12_ | 7.95E+06 | 1273999 | 1.01E+07 | 2424486 | 0 | 0 | 0.006 | 3.28 |
| Maltol | C_6_H_6_O_3_ | 4.19E+06 | 3828083 | 7.65E+06 | 2819499 | 0 | 0 | 0.03 | 1.49 |

* Values of this table are the absolute intensity normalized and scaled using pareto scaling.

# Calculated using t-test, tails 2, type 3 between the control values and the samples.

| **Table S3**. Mass spectrometry results of the annotated compounds from the needle extracts identified by DI-ESI-HRMS. | | | | | | | | | |
| --- | --- | --- | --- | --- | --- | --- | --- | --- | --- |
| Compound | Molecular Formula | Control* (average) | Control*  SD (±) | Sample* (Average) | Sample*  SD (±) | Blank*  (Methanol average) | Blank*  SD (±)  (Methanol) | *p-*value# | Ratio CTR/SA |
| 3-Dehydroshikimic acid | C_7_H_8_O_5_ | 28083632 | 7116968 | 111226972 | 2929995 | 0 | 0 | 3.17E-05 | 0.25 |
| 3-Hexenedioic acid | C_6_H_8_O_4_ | 13562843 | 3046307 | 3853589 | 79433.74638 | 871192 | 1006258 | 0.03 | 3.52 |
| 3-Oxoanticopalic Acid | C_20_H_30_O_3_ | 3386369 | 1163594 | 10395213 | 1034269 | 0 | 0 | 0.0002 | 0.33 |
| 4-O-Methyl-D-glucaric acid | C_7_H_12_O_8_ | 77996410 | 19924219 | 31896094 | 1240494 | 0 | 0 | 0.05 | 2.45 |
| 5-Dehydro-4-deoxy-D-glucaric acid | C_6_H_8_O_7_ | 129332882 | 54449761 | 23538205 | 596040 | 0 | 0 | 0.08 | 5.49 |
| Andrographolide | C_20_H_30_O_5_ | 29970044 | 10682102 | 58057470 | 4655350 | 0 | 0 | 0.02 | 0.52 |
| Ascaridole | C_10_H_16_O_2_ | 7376638 | 5247757 | 95425076 | 16377141 | 3203901 | 770895 | 2.63E-05 | 0.08 |
| Ascorbic Acid | C_6_H_8_O_6_ | 9499994 | 2960786 | 12560682 | 420787 | 0 | 0 | 0.21 | 0.76 |
| Benzoic acid | C_7_H_6_O_2_ | 50176596 | 9398120 | 6727042 | 496390 | 488228 | 976457 | 0.02 | 7.46 |
| Caproic acid | C_6_H_12_O_2_ | 6378064 | 3258339 | 10443596 | 1274547 | 0 | 0 | 0.15 | 0.61 |
| Cellobiose | C_12_H_22_O_11_ | 63125736 | 13389184 | 11106527 | 499486 | 805205 | 1610411 | 0.02 | 5.68 |
| Citronellic acid | C_10_H_18_O_2_ | 2746811 | 2810031 | 8632782 | 815704 | 3479411 | 917478 | 0.053 | 0.32 |
| Coniferyl alcohol | C_10_H_12_O_3_ | 7677456 | 1605051 | 15985241 | 717324 | 4985125 | 1036746 | 0.001 | 0.48 |
| Coumaric acid | C_9_H_8_O_3_ | 30769724 | 5080238 | 1830707 | 608631 | 0 | 0 | 0.009 | 16.81 |
| Dehydroabietic acid | C_20_H_28_O_2_ | 4088702 | 1765561 | 11965314 | 943956 | 5770572 | 981754 | 0.002 | 0.34 |
| Dehydroascorbic acid | C_6_H_6_O_6_ | 8355791 | 2809930 | 10310075 | 137330 | 0 | 0 | 0.35 | 0.81 |
| Dehydropinifolic acid | C_20_H_30_O_4_ | 19399590 | 6822460 | 41317214 | 3258724 | 0 | 0 | 0.008 | 0.47 |
| Deoxyquinic acid | C_7_H_12_O_5_ | 22670721 | 5832444 | 34881389 | 849251 | 5013189 | 1004843 | 0.06 | 0.65 |
| D-Glucose | C_6_H_12_O_6_ | 55574456 | 37576451 | 3509846 | 381674 | 2861872 | 4551151 | 0.14 | 15.83 |
| D-Glucuronic acid methyl ester | C_7_H_12_O_7_ | 94615906 | 22709205 | 98107920 | 4294157 | 0 | 0 | 0.82 | 0.96 |
| D-hexose | C_6_H_12_O_6_ | 1094267776 | 93232072 | 53779279 | 2734344 | 9494271 | 6534061 | 0.003 | 20.35 |
| Dihydrosinapyl alcohol | C_11_H_16_O_4_ | 16045472 | 5079647 | 76909220 | 10661606 | 44860960 | 11002879 | 8.22E-06 | 0.21 |
| Disaccharide (unidentified) | C_13_H_22_O_11_ | 448454058 | 144586377 | 15722495 | 828562 | 0 | 0 | 0.04 | 28.52 |
| D-Xylose | C_5_H_10_O_5_ | 35996905 | 1868229 | 7111626 | 265319 | 0 | 0 | 0.0006 | 5.06 |
| Galactosyl pinitol | C_13_H_24_O_12_ | 1228410688 | 262598209 | 20318393 | 455865 | 0 | 0 | 0.02 | 60.46 |
| Galactosylglycerol | C_9_H_18_O_8_ | 107634917 | 36251079 | 2940716 | 294414 | 3396025 | 1338973 | 0.04 | 36.60 |
| Gallic Acid | C_7_H_6_O_5_ | 4667713 | 789490 | 7154094 | 729727 | 1578371 | 1053654 | 0.007 | 0.65 |
| Glucoheptonic acid | C_7_H_14_O_8_ | 1013381888 | 374474967 | 21790401 | 689458 | 4796425 | 1513481 | 0.04 | 46.51 |
| Gluconic Acid | C_6_H_12_O_7_ | 598491008 | 172586460 | 608466716 | 30325812 | 1466680 | 1759603 | 0.93 | 0.98 |
| Gluconolactone | C_6_H_10_O_6_ | 76390440 | 7494093 | 48301208 | 2278349 | 0 | 0 | 0.008 | 1.58 |
| Glucuronic acid | C_6_H_10_O_7_ | 109990861 | 22175437 | 46069323 | 2136212 | 0 | 0 | 0.04 | 2.39 |
| Hydroxycitronellal | C_10_H_20_O_2_ | 36122344 | 15724387 | 79864548 | 17214052 | 135552944 | 22694672 | 0.01 | 0.45 |
| Hydroxyoxodehydroabietic acid | C_20_H_26_O_4_ | 3343258 | 1245686 | 10238040 | 796568 | 0 | 0 | 0.0004 | 0.33 |
| Imbricatolic Acid | C_20_H_34_O_3_ | 979726 | 1696935 | 15504307 | 1588869 | 0 | 0 | 1.25E-05 | 0.06 |
| Isocupressic acid | C_20_H_32_O_3_ | 3721335 | 1481504 | 8062747 | 981140 | 0 | 0 | 0.01 | 0.46 |
| Lambertiainc Acid | C_20_H_28_O_3_ | 16046378 | 5124415 | 51975365 | 3873926 | 1083394 | 1259497 | 5.83E-05 | 0.31 |
| Lauric acid | C_12_H_24_O_2_ | 22349515 | 8823438 | 45495802 | 5044608 | 77691918 | 15314018 | 0.02 | 0.49 |
| Lepidimoide acid | C_12_H_18_O_10_ | 30729008 | 7708364 | 44334590 | 2227236 | 0 | 0 | 0.07 | 0.69 |
| Methyl jasmonate | C_13_H_20_O_3_ | 21534161 | 10546600 | 56088776 | 2721640 | 8870456 | 1655697 | 0.02 | 0.38 |
| Methyl pinifolate | C_21_H_34_O_4_ | 15587499 | 6905196 | 25034023 | 1664306 | 0 | 0 | 0.13 | 0.62 |
| Methyl-D-glucoside | C_7_H_14_O_6_ | 116322382 | 61989239 | 8505648 | 676540 | 0 | 0 | 0.1 | 13.68 |
| Myristic acid | C_14_H_28_O_2_ | 29355166 | 9015039 | 71283401 | 9579315 | 104248808 | 25382294 | 0.001 | 0.41 |
| Myrtenyl isovalerate | C_15_H_24_O_2_ | 1860813 | 1611513 | 7606387 | 1149895 | 4417440 | 1151909 | 0.006 | 0.24 |
| Nonanoic acid | C_9_H_18_O_2_ | 238580965 | 106985214 | 403696145 | 27198799 | 250579924 | 36325929 | 0.11 | 0.59 |
| Octose | C_8_H_16_O_8_ | 661227285 | 163546140 | 14110741 | 789714 | 6933758 | 2087220 | 0.02 | 46.86 |
| Palmitic acid | C_16_H_32_O_2_ | 413069941 | 136065796 | 984220707 | 169803781 | 642711648 | 1.31E+08 | 0.002 | 0.42 |
| Pentadecanoic acid | C_15_H_30_O_2_ | 15616833 | 4604896 | 38205812 | 5122611 | 59029981 | 15395265 | 0.0007 | 0.41 |
| Perillic acid | C_10_H_14_O_2_ | 3986766 | 2442461 | 9026171 | 1022841 | 2822424 | 759773 | 0.05 | 0.44 |
| Pinifolic Acid | C_20_H_32_O_4_ | 36132348 | 13104067 | 80926491 | 4795190 | 426674 | 853349 | 0.01 | 0.45 |
| Pinifolic acid derivative | C_20_H_32_O_5_ | 20127767 | 6488427 | 75941305 | 4725175 | 0 | 0 | 1.51E-05 | 0.27 |
| Pinifolic acid derivative | C_20_H_34_O_4_ | 3261701 | 742474 | 36931157 | 2676190 | 0 | 0 | 9.75E-07 | 0.09 |
| Pinonic acid | C_10_H_16_O_3_ | 23283736 | 7136618 | 59637430 | 2987947 | 46090625 | 6888186 | 0.002 | 0.39 |
| Pinusolidic acid | C_20_H_28_O_4_ | 7708158 | 2973674 | 50622033 | 3604223 | 0 | 0 | 8.66E-07 | 0.15 |
| Quinic acid | C_7_H_12_O_6_ | 3430975232 | 967104607 | 2097615374 | 90061050 | 932142 | 1082756 | 0.14 | 1.64 |
| Resin acid derivative | C_21_H_32_O_4_ | 5035056 | 1822911 | 12040469. | 933958 | 0 | 0 | 0.004 | 0.42 |
| Salicylic acid | C_7_H_6_O_3_ | 21535180 | 1324008 | 455911 | 355994 | 0 | 0 | 0.0003 | 47.24 |
| Sebacic Acid | C_10_H_18_O_4_ | 25538348 | 7789768 | 35245890 | 2324110 | 12689688 | 2517361 | 0.15 | 0.72 |
| Shikimic acid | C_7_H_10_O_5_ | 491879477 | 129441786 | 360480369 | 15444836 | 8851933 | 902512 | 0.22 | 1.36 |
| Sinapaldehyde | C_11_H_12_O_4_ | 302531664 | 135526555 | 776089884 | 31200844 | 18012985 | 5068866 | 0.01 | 0.39 |
| Sorbitol | C_6_H_14_O_6_ | 31556664 | 3791430 | 32825985 | 1683978 | 1078098 | 1263297 | 0.69 | 0.96 |
| ß-Caryophyllonic acid | C_15_H_24_O_3_ | 26386510 | 5628266 | 56265628 | 3430137 | 14498370 | 3468065 | 0.0004 | 0.47 |
| ß-Methylionone | C_14_H_22_O | 24903748 | 13523741 | 94207870 | 5304416 | 226149288 | 37841632 | 0.002 | 0.26 |
| Stearic Acid | C_18_H_36_O_2_ | 298611482 | 112083919 | 689888266 | 124268285 | 428566840 | 82464625 | 0.005 | 0.43 |
| Tachioside | C_13_H_18_O_8_ | 150770941 | 39648160 | 2996735 | 120389 | 0 | 0 | 0.02 | 50.31 |
| Vanillic Acid | C_8_H_8_O_4_ | 28859048 | 3185442 | 6238383 | 44163 | 0 | 0 | 0.006 | 4.63 |
| Vanillin | C_8_H_8_O_3_ | 9464607 | 1507515 | 8908660 | 165589 | 3678112 | 262651 | 0.59 | 1.06 |

*Values of this table are the absolute intensity normalized and scaled using pareto scaling.

# Calculated using t-test, tails 2, type 3 between the control values and the samples.

| **Table S4.** Mass spectrometry results of the annotated compounds of the needle extracts identified by DI-APPI-HRMS. | | | | | | | | | |
| --- | --- | --- | --- | --- | --- | --- | --- | --- | --- |
| Compound | Molecular Formula | Control* (average) | Control * SD (±) | Sample* (average) | Sample* SD (±) | Blank*  (methanol average) | Blank*  SD (±)  (Methanol) | *p*-value# | Ratio  CTR/SA |
| 12-Hydroxyabietic acid | C_20_H_30_O_3_ | 3514420 | 1264437 | 7912233 | 1381253 | 0 | 0 | 9.4E-06 | 0.16 |
| 15-Hydroxy-7-oxodehydroabietic acid | C_20_H_26_O_4_ | 8849098 | 2005778 | 13548543 | 2418517 | 0 | 0 | 0.0006 | 0.15 |
| 15-Hydroxydehydroabietic acid | C_20_H_28_O_3_ | 22326124 | 7304658 | 28403604 | 7001821 | 0 | 0 | 0.11 | 0.26 |
| 5-hydroxyferulic acid | C_10_H_10_O_5_ | 8920193 | 7162871 | 1828847 | 1007697 | 0 | 0 | 0.04 | 3.92 |
| 7-Hydroxy-4-methyl-3-propyl-2H-chromen-2-one | C_13_H_14_O_3_ | 106682997 | 75894138 | 6456669 | 841515 | 0 | 0 | 0.01 | 11.75 |
| 8-Prenylnaringenin | C_20_H_20_O_5_ | 10849196 | 3021725 | 0 | 0 | 0 | 0 | 7.76E-05 | - |
| Abietic acid | C_20_H_30_O_2_ | 4328952 | 1369723 | 8214615 | 2017007 | 0 | 0 | 0.0003 | 0.17 |
| Allohydroxymatairesinol | C_19_H_20_O_6_ | 9635869 | 3992497 | 236875 | 749066 | 0 | 0 | 0.0007 | 16.85 |
| alpha-irone | C_14_H_22_O | 8798723 | 1396873 | 7616730 | 1660288 | 0 | 0 | 0.13 | 0.18 |
| Amylcinnamaldehyde | C_14_H_18_O | 2204862 | 2261620 | 7768791 | 4068922 | 0 | 0 | 0.003 | 0.29 |
| Artemisinic acid | C_15_H_22_O_2_ | 31649574 | 10010804 | 49466426 | 9979339 | 0 | 0 | 0.003 | 0.20 |
| Ascaridole | C_10_H_16_O_2_ | 8081685 | 2703544 | 47624586 | 23048539 | 0 | 0 | 0.0004 | 0.06 |
| Calamenene | C_15_H_22_ | 90073348 | 14757895 | 85184356 | 32073560 | 0 | 0 | 0.68 | 0.17 |
| Caryophyllene | C_15_H_24_ | 13859867 | 4045431 | 8426651 | 2894894 | 0 | 0 | 0.01 | 0.48 |
| Caryophyllene oxide | C_15_H_24_O | 70618995 | 20503913 | 89943442 | 21971936 | 434225 | 868450 | 0.09 | 0.23 |
| Dehydroabietic acid | C_20_H_28_O_2_ | 4662344 | 866744 | 7837089 | 1542613 | 0 | 0 | 8.21E-05 | 0.11 |
| Dehydropinifolic acid | C_20_H_30_O_4_ | 7582695 | 2013483 | 12881335 | 2008897 | 0 | 0 | 0.0001 | 0.16 |
| Demethoxylpinoresinol | C_19_H_20_O_5_ | 232066638 | 43601755 | 218927 | 692310 | 0 | 0 | 8.03E-06 | 199.16 |
| Demethoxypinoresinol | C_19_H_20_O_5_ | 13154835 | 3958060 | 0 | 0 | 0 | 0 | 0.0001 | - |
| Gibberellin A120 | C_19_H_22_O_4_ | 6464005 | 1495043 | 10011396 | 1999437 | 0 | 0 | 0.0008 | 0.15 |
| Gibberellin A9 | C_19_H_24_O_4_ | 8591037 | 2681777 | 11769128 | 1389282 | 0 | 0 | 0.02 | 0.23 |
| Hydroxy-beta-ionone | _C13H20O2_ | 49806609 | 8469083 | 79222493 | 16030397 | 0 | 0 | 0.0002 | 0.11 |
| Lariciresinol | C_20_H_24_O_6_ | 36826314 | 9485214 | 0 | 0 | 0 | 0 | 4.97E-05 | - |
| Ligballinol | C_18_H_18_O_4_ | 28314079 | 9450851 | 245776 | 777214 | 0 | 0 | 0.0002 | 38.45 |
| Lignan 1 | C_20_H_22_O_5_ | 96301948 | 40149543 | 2054487 | 761420 | 0 | 0 | 0.0008 | 19.54 |
| Lignan 2 | C_19_H_22_O_5_ | 9982314 | 1689055 | 0 | 0 | 0 | 0 | 4.33E-06 | - |
| Myrtenyl acetate | C_12_H_18_O_2_ | 4495502 | 527380 | 10368791 | 1891260 | 0 | 0 | 1.59E-06 | 0.05 |
| Myrtenyl isovalerate | C_15_H_24_O_2_ | 10922991 | 3010910 | 33847579 | 6141312 | 0 | 0 | 8.48E-08 | 0.09 |
| Nootkatone | C_15_H_22_O | 33992340 | 7614168 | 63855070 | 13111318 | 0 | 0 | 3.11E-05 | 0.12 |
| Perillic acid | C_10_H_14_O2 | 4338537 | 1624860 | 8791210 | 2918827 | 0 | 0 | 0.001 | 0.18 |
| Pinoresinol | C_20_H_22_O_6_ | 8242448 | 1724992 | 0 | 0 | 0 | 0 | 1.5E-05 | - |
| Pinosylvin | C_14_H_12_O_2_ | 11080161 | 1072511 | 7461090 | 1800289 | 451972.5 | 903945 | 0.0001 | 0.14 |
| Pinusolidic acid | C_20_H_28_O_4_ | 19322517 | 6802813 | 24551495 | 2852759 | 0 | 0 | 0.09 | 0.28 |
| Sesquiterpene derivative | C_15_H_20_ | 9947784 | 2446614 | 17439128 | 3372068 | 0 | 0 | 8.88E-05 | 0.14 |
| Simonellite | C_19_H_24_ | 9122616 | 2609981 | 22925879 | 6264482 | 0 | 0 | 3.21E-05 | 0.11 |
| Styryl lactone | C_13_H_12_O_2_ | 988733979 | 220576739 | 91085413 | 27489623 | 21924858.3 | 12415806.8 | 3.39E-05 | 2.42 |
| Taxiresinol | C_19_H_22_O_6_ | 134365987 | 58588058 | 202195 | 639398 | 0 | 0 | 0.0009 | 289.76 |
| Tetramethoxystilben-2-ol | C_18_H_20_O_5_ | 199101426 | 45096236 | 0 | 0 | 0 | 0 | 2.37E-05 | - |
| Trimethyleugenol | C_13_H_18_O_2_ | 56073485 | 5326887 | 94645938 | 35284514 | 0 | 0 | 0.007 | 0.05 |
| z-Bornyl p-coumarate | C_19_H_24_O_3_ | 26726990 | 3910016 | 51777147 | 7095677 | 0 | 0 | 1.72E-07 | 0.08 |

* Values of this table are the absolute intensity normalized and scaled using pareto scaling.

# Calculated using t-test, tails 2, type 3 between the control values and the samples.

| **Table S5**. Secreted proteins of *Phacidium infestans* implicated in plant cell wall degradation as annotated by dbCAN3. | | | | |
| --- | --- | --- | --- | --- |
| Protein | **CAZy family** | **Blastp ID** | **Known substrate^#^** | **Percentage ID / organism** |
| [Phain-OT5-proseq9131](https://bcb.unl.edu/dbCAN2/domain.php?jobid=2023111560349&gene=g9131) | GH11 | Endo-1.4-beta-xylanase 11A | Hemicellulose | 95.07 % *L.hyalina* |
| [Phain-OT5-proseq3700](https://bcb.unl.edu/dbCAN2/domain.php?jobid=2023111560349&gene=g3700) | GH115 | Gylcosyl hydrolase 115 C-terminal domain-containing protein | Hemicellulose | 91.9 %. *L.subtilissima* |
| [Phain-OT5-proseq2568](https://bcb.unl.edu/dbCAN2/domain.php?jobid=2023111560349&gene=g2568)  Phain-OT5- proseq3722  Phain-OT5- proseq5359 | GH27 | Alpha-galactosidase  Putative alpha-galactosidase  Putative alpha-galactosidase D | Hemicellulose | 98.99 % *L.subtilissima*  99.6 % *L. subtilissima*  95.12 % *L.* *hyalina* |
| [Phain-OT5-proseq10364](https://bcb.unl.edu/dbCAN2/domain.php?jobid=2023111560349&gene=g10364) | GH31 | Putative alpha/beta-glucosidase | Hemicellulose | 95.88 % *L.subtilissima* |
| [Phain-OT5-proseq6458](https://bcb.unl.edu/dbCAN2/domain.php?jobid=2023111560349&gene=g6458)  Phain-OT5-proseq6940 | GH35 | Putative beta-galactosidase C  Putative beta-galactosidase A | Hemicellulose | 94.57 % *L.subtilissima*  97.52% *L.* *hyalina* |
| [Phain-OT5-proseq5262](https://bcb.unl.edu/dbCAN2/domain.php?jobid=2023111560349&gene=g5262) | GH51 | Alpha-L-arabinofuranosidase A | Hemicellulose | 97.53 % *L.subtilissima* |
| [Phain-OT5-proseq8821](https://bcb.unl.edu/dbCAN2/domain.php?jobid=2023111560349&gene=g8821) | GH54+CBM42 | Putative alpha-L-arabinofuranosidase B | Hemicellulose | 98.03 % *L. subtilissima* |
| [Phain-OT5-proseq8264](https://bcb.unl.edu/dbCAN2/domain.php?jobid=2023111560349&gene=g8264) | GH62 | Alpha-L-arabinofuranosidase | Hemicellulose | 97.19 % *L. hyalina* |
| [Phain-OT5-proseq1022](https://bcb.unl.edu/dbCAN2/domain.php?jobid=2023111560349&gene=g1022)  Phain-OT5-proseq8746 | GH93 | Checkpoint serine/threonine-protein kinase  Protoheme IX farnesyltransferase. mitochondrial | Hemicellulose | 79.9 % [*L. willkommii*](https://www.uniprot.org/taxonomy/215461)  79.73% *L.subtilissima* |
| [Phain-OT5-proseq2613](https://bcb.unl.edu/dbCAN2/domain.php?jobid=2023111560349&gene=g2613) | GH43 | Putative arabinan endo-1.5-alpha-L-arabinosidase A | Hemicellulose + pectin | 95.67 % *L. subtilissima* |
| [Phain-OT5-proseq10384](https://bcb.unl.edu/dbCAN2/domain.php?jobid=2023111560349&gene=g10384)  [Phain-OT5-proseq10480](https://bcb.unl.edu/dbCAN2/domain.php?jobid=2023111560349&gene=g10480)  [Phain-OT5-proseq3158](https://bcb.unl.edu/dbCAN2/domain.php?jobid=2023111560349&gene=g3158)  Phain-OT5-proseq[3647](https://bcb.unl.edu/dbCAN2/domain.php?jobid=2023111560349&gene=g3647)  [Phain-OT5-proseq 401](https://bcb.unl.edu/dbCAN2/domain.php?jobid=2023111560349&gene=g401)  Phain-OT5-proseq667  [Phain-OT5-proseq9596](https://bcb.unl.edu/dbCAN2/domain.php?jobid=2023111560349&gene=g9596)  [Phain-OT5-proseq11251](https://bcb.unl.edu/dbCAN2/domain.php?jobid=2023111560349&gene=g11251)  Phain-OT5-proseq6681  Phain-OT5-proseq1075  Phain-OT5-proseq1809 | GH28 | Endo-polygalacturonase  Polygalacturonase  Putative galacturan 1.4-alpha-galacturonidase C  Polygalaturonase  Exopolygalacturonase X  Putative endopolygalacturonase  Alpha-L-rhamnosidase  Putative rhamnogalacturonase A  Alpha-L-rhamnosidase  Putative endopolygalacturonase  Rhamnogalacturonase A | Pectin | 95.5 % *L. subtilissima*  99.20 % *L. subtilissima*  96.9 % *L. subtilissima*  98.22 % *L.subtilissima*  94.94 % *L. subtilissima*  96.9 % *L. subtilissima*  95.64 % *L. subtilissima*  98.29 % *L. subtilissima*  86.52 % *L. subtilissima*  95.73 % *L. subtilissima*  99.10 % *L. subtilissima* |
| [Phain-OT5-proseq3871](https://bcb.unl.edu/dbCAN2/domain.php?jobid=2023111560349&gene=g3871)  Phain-OT5-proseq8712 | GH78 | Alpha-L-rhamnosidase six-hairpin glycosidase domain-containing protein  Alpha-L-rhamnosidase six-hairpin glycosidase domain-containing protein | Pectin | 94.5% *L. hyaline*  98.40% *L.subtilissima* |
| [Phain-OT5-proseq2544](https://bcb.unl.edu/dbCAN2/domain.php?jobid=2023111560349&gene=g2544)  Phain-OT5-proseq[7818](https://bcb.unl.edu/dbCAN2/domain.php?jobid=2023111560349&gene=g7818)  Phain-OT5-proseq[913](https://bcb.unl.edu/dbCAN2/domain.php?jobid=2023111560349&gene=g913)  Phain-OT5-proseq3538 | PL1 | Putative pectate lyase A  Putative pectate lyase C  Putative pectin lyase A  Putative pectate lyase A | Pectin | 91.98% *L.subtilissima*  75.4%. *L.*[*suecica*](https://www.uniprot.org/taxonomy/602035)  92.13% *L.hyalina*  98.78% *L.subtilissima* |
| [Phain-OT5-proseq10047](https://bcb.unl.edu/dbCAN2/domain.php?jobid=2023111560349&gene=g10047)  Phain-OT5-proseq1538 | GH12 | Xyloglucan-specific endo-beta-1.4-glucanase  Putative xyloglucan endohydrolase A | Cellulose | 74.41% *L. cervine*  87.95% *L. arida* |
| [Phain-OT5-proseq3246](https://bcb.unl.edu/dbCAN2/domain.php?jobid=2023112010748&gene=g3246)  Phain-OT5-proseq[4170](https://bcb.unl.edu/dbCAN2/domain.php?jobid=2023112010748&gene=g4170)  Phain-OT5-proseq7132 | GH7 | Endoglucanase 7a  Glucanase  Putative 1.4-beta-D-glucan cellobiohydrolase B | Cellulose | 99.12% *L. subtilissima*  99.1% *L. subtilissima*  99.78% *L. subtilissima* |
| [Phain-OT5-proseq6582](https://bcb.unl.edu/dbCAN2/domain.php?jobid=2023112010748&gene=g6582) | GH74 | Xyloglucanase | Cellulose | 95.68% *L. subtilissima* |

# retrieved from CAZY **(Zhao et al., 2014)**.

**Additional file 3**

The predicted protein sequences used in the study are listed below.

> Phain-OT5-proseq999

MVFPAIAERIARGPRATEFSEPSSLMEIETHKILAWVFSTDGILALILAHTAFRFSTTQMPRPIETRPVRVANCSGYHGDPAFEMYRQATLGDVDFITGDYIAEVNLANNAQAWRNGKHPGYEPTAWDGLQQTIDVISQKGIRVVINGGALDPKALALKVDALASEKNLNLRVAYLSGDDLYPILGPNMPTTKEELRHLDSGNPSAAPDELTYAFLNNGGPAVSMVSAHAYLGARGIVDGLRRGADIIICGRVADASPVIAAAWFWHNWSDENYDKLAGSLIAGHLIECSAYVTGGNFSGFDRYDLDNLVAPGFPIAEIATDGTCVIAKHSGTDGFINVDTVRCQFLYELQGNTYLNSDVSAHIADLHIENAGEDRVRVSGIRGSPPPSTTKCAIFYQGGYEAQLLLNATGYATANKWDLLEKQLRRFIPAEVLKDLETLEFQRVGVPAQNPSSQLESTTYLRVFITSRDEKAVAAVVKAMGNISLKHFSGFHASLDMRTAIPRPFLAYYPAMIKQSNINEAINFIDGPNKISSFDTGHPPRYEELGIRDTYDPKDPQIHNCSNQQMRLGDIALGRSGDKGGNLNVGFFPLNTAHWPWLRDYMTKDRMRQLLGKDWHDSFFLERVEFPHIHAVHFVVYGILGRGVSSSSRLDGFGKGFVDYVRDKFVEVPVDIL

> Phain-OT5-proseq3542

MEPALNISSVNDEKSLSSQSSLRKPQLDPLCHILTPIGMLGYGFDEALTHQALQDLKNSNVPTALILDSGSTDSGPAKLALGIMTSPRSSYERDFTKLLRLANTFHVPLLISSAGGDGSDVHVDEFLDIVREICEKGNDSYKFKTLAIYSNVSKAKVSQSLHAGEVEGCGASVPELTDSDIECAATIVAQMGPEPFVQAMEAEPDFDIIIGGRAYDPSPYVAFCIFQAEKVLAASGKTLTAKQLGGFTHMGKIMECGAICATPKSASAMATVYQDGTFDIKPLNADARCTPSSVAAHTLYEKSRPDILSGPGGNLNLTHSMYAQLPDQRTVRVHGATFQFSRDVNLPYTVKLEAAKIVGHRTIFMGGIRDPILISQIPSFQSRIQEYVATQNPSSSPSSGEFWKLGFHTYGANGIMGAMEPGDSTFQPREIFIVGEALASSQKLATSIAATARVAAAHGAYPGQRGTGGNFAMGVGGKLEVEMGPCAEFCIYHLMPLLVGEEGAKKIGDNSMTSKSDPSTLPIFSWKMSVIGKGDLAPKAPIRSSPATALDPPRQNPRPSQPSTLNLTSPLTLTDIAPVIRSKNSGPYEITLDVVFSSLPIYAIIKASSLLTPATLARMYHLNEADIVWCGFFDQAMAWKCTLPRRGNAGERRVAGGFMEGDVHASQQYAGLLGLELGEEVRAEIRGLGLGVVKLRFRDDRALSVGKLKKVIGGIEYARKHLPSTLEPLAIPGAQPQVTVDTL

> Phain-OT5-proseq5913

MAATETEPHHKTYAPVATQPGLISRILSNSSVLTGRNSQREYDDSDEDEHIISMASDWKMMPELRMIQRQGEKDEAKGRKLGVTWNNLTIKGIGADAAINENVGSQFNIPQAIMEARGGAPLKTILENSHGCVKPGEMLLVLGRPGAGCTSLLKVLANRREGFAEVTGDVHWGAMNPKDAAQYRGQIVMNTEEELFFPTLTVGQTIDFATALKIPFNLPSDKATPADFQKENVDFLLKSLGIPHTRDTKVGNEFVRGVSGGERKRVSILETLATRASVFCWDNSTRGLDASTALEYTKAIRAITDIFGLASIVTLYQAGNGIYNLFDKAMVIDEGKQIYYGPLKQARPFMEDLGFYCDDGANVADFLTGVTVPSERRLREGVESFPRTADEIRAAYEKSSIKKEMEAEYNYPTSEEAISRTEDFRIGVQHEKAKSLSKKSPYTVSFGRQIDACIRRQYQILWGDKASLIIKQSSVLIQALIAGSLFYNAPANSGGLFLKSGALFLSLLFNSLMAMSEVTESFSGRPVLAKHKDFALFHPAAFCLAQVAADVPVILFQISHFSLVLYFMVGLRQDAGAFFTFWIFLFATSMCMTALFRSIGAAFSTFDGASKVSGFLVSALIMYTGYMIQKPAMHPWFVWIYWIDPLAYGFSGILANEFKDAIIPCVGINLVPNGPQYGDLLYSACAGVGGARPGATSVTGGEYLASLSYSASNIWRNFGIVWAFWVLFVAITIVATIHWKAHGGKGGVLLIPREKAKKNTHHLVADEESQSKVQVDQEKKVAGSGASSSDETTVTDQQLVRNTSVFTWKNLSYTVKTPSGDRTLLDDVHGWVKPGQLGALMGSSGAGKTTLLDVLAQRKTDGTIKGSILVDGRPLTVSFQRSAGYCEQLDVHEPLATVREALEFSALLRQSRTTPREEKLKYVDSIINLLEMHDIENTLIGSTGSGLSVEQRKRLTIGVELVSKPSILIFLDEPTSGLDGQAAFNIVRFLRKLADIGQAVLVTIHQPSAQLFLQFDSLLLLARGGKTVYFGDIGPSAATLKEYFARYDAPCPKDANPAEHMIDVVSGSHSQTKDWSQVWLDSPEYKRSIEELEHITSDAASKPPGTVDDGFEFAMPLWEQTKIVTHRMNISVWRNTDYVNNKLALHVGSALFNGFSFWQIGHSIADLQLRLFSVFSFIFVAPGVIAQLQPLFIDRRDIYEAREKKSKMYSWIAFVTGLIISELPYLCVCAVLYFVCWYYTVGFPTDSNKAGATLFVMIMYEFVYTGIGQFIAAYAPNAVFATLANPLVIGTLVGFCGVLVPYAQITAFWRYWIYWLNPFNYLMGSLLTFTSFSAPVHCAELEFAIFNPPLNQTCGVYLADYMGGMGARTNLTNPDDRSHCKVCEYRTGSDYLTSLNLADYYYGWRDAGIVVLFALVGYAMVYMLMKLRTKQSKKAE

> Phain-OT5-proseq8177

MSDKETYQEEAPRALENSPNAPTSPYSTARTSADTVADPEVQRENARHNNPNGFARTESGVNVKSAEAEFATLQRELTGISQTSRHLSRTHSRQSQASKTKGSGDVEKVATSESNSEGEQFDLESTLRGNQNAERDAGIRSKHIGVIWDGLTVTGAGGVTNFVKTFPDAFVSFFNVIETGMNILGIGKKGRDVKILDDFRGVVTPGEMVLVLGRPGSGCTTFLKVIANQRFGYTGVDGEVLYGPFDAKTFAKQYRGEAVYNQEDDVHHPTLTVGQTLGFALDTKTPGKRPDGLSKADFKDKVITTFLRMFNIEHTRNTIVGNQFVRGVSGGERKRVSIAEMMVTSATLCAWDNSTRGLDASTALDYAKSLRVMTNIYKTTTFVSLYQASENIYKQFDKVLVIDDGREVYFGPAKEARSYFEGLGFKEKPRQTSPDFLTGCTDEFEREYADGRTSENAPHSPETLAKAFNDSKYAKMLDDEMVQYRKTLAEDKQRQEDFQVAVHDSKRRGASKKSVYSIPFFLQVWTLMQRQYLIKWQDKFSLVVSWITSIVIAIVLGTVWLDLPKTSAGAFTRGGLLFISLLFNAFQAFGELASTMIGRPIVNKHKAYTFHRPSALWIAQILVDVAFSAIQIFFFSVIVYFMCGLVRDAGAFFTFYLVIVSGYLAMTLFFRTIGCVCPDFDYAIKFAATIITFFVITSGYLIQYQSEKVWLRWIYWINALGLGFAALMENEFSRIDLTCTSESLVPSGPGYNNISHQVCTLPGSVSGTNTVSGSAYITKGFDYPPSELWRNWGIILVLIVGFLFSNAMLGEWINFGAGGNTAKVFQKPNKEREELNAALVARREARRSSKNEAEGSELNINSKAILTWEGLKYDVPTPAGQLRLLNNIYGYVQPGELTALMGASGAGKTTLLDVLASRKNIGVISGDVLVDGIKPGTSFQRGTSYAEQLDVHEPTQTVREALRFSADLRQPIEVPQSEKYAYVEEVLSLLEMEDMADAIIGDPESGLAVEQRKRVTIGVELAAKPELLLFLDEPTSGLDSQSAFNIVRFLKKLANAGQAILCTIHQPNAALFENFDRLLLLQKGGQTVYFGDIGKDAVVLLDYLSRHGAHCPHDANPAEYMLDAIGAGQAPRVGDRDWGDIFAESPELANIKDRISQMKAKRLEAVGSHAKVDEKEFATPLLHQLKTVQKRTNLSFWRSPNYGFTRLFNHVIISIITGLAYLHLDNSRASLQFRVFVIFQVTVLPALILAQVEPKYAISRMIFYREASSKMYGQFAFASSLVVAEMPYSILCAVGFFLPLYYMPGFQHASSRAGYQFLMILITELFSVTLGQMVAAITPSPFISALLNPFIIITFALFCGVTIPKAQIPKFWRAWLYQLDPFTRLIGGMVVTELHERPVICTPSEFNRFSSPPDQNCGDYMKPFFKASGAGYIADNATRACEYCAYKMGDQFYTGLDLNFDNRWRDMGIFLAFVGSNLILLFLGSRYLNFNRR

> Phain-OT5-proseq7851

MFTENAEGREGTRVPLHNTDNPSERSQNPEEANSSDSDVNNDGTWGERDVGGPVNFRNAMLDYEDMRRELTSLSKTRTGKSGKSGKSGKSDRKRSFGLNRGFTNRSHQTNAQFEPERDAADEVQESDEDDDEEEDDFELRDFLKDGHFEKRQEGRSAKKVGVLYKNLTVQGVGATSTFVKTLPSAVIGTFGPDLYNLLSRCIPFLPIPGASGGKRNLIHDFTGVVRDGEMLLVLGRPGSGCSTFLKAVANKRGAFAGVDGEVSYGGIPADEQRKSYRGEVNYNEEDDQHFPTLTVEQTLDFSLLNKTRKYEKGDIPIIIGALLKMLSFEDISGGERKRVSIAETLATKSTVVAWDNSTRGLDASTALDYAKSLRVMTDVSNRTTLVTLYQAGEGIYKVMDKVLVIDGGRMMYSGPAGEAKKYFHDLGYHCPERQTTADFLTACTDPTERRFRDDFEGPIPKGPVELEQAFRESDAYKRLLQDVESYEKMLKETDHADAREFKQSVKESKSKTVSNRSSYTVSFFRQVLACTKREFWLTWGDKTTLYTKFFIIISNGLIVGSLFYGQTTDTEGSFTRGGTLFFSILFLGWLQLSELMKAVSGRTIISRHNDYAFYRPSAVVIARVLQDFPLILAQVIPFAIIMYFITGLDVDASKFFIYFLFIYTTTICITALYRMFAALSPAIDDAVRFSGVGLNLMIVYTGYVIAKPQLLSEYIWFGWIYYINPLSYSFEAVLSNEFYNKVLACAPSQTVPRGPGFDNPAYQGCAFTGAQIGSLNIPGSTYLNSGFEYSRGHLWRNFGVIIAFAVLYILITAVATELFDFTSGGGGALEYKRSKAAKQKVKAALAPTDIEKGPREPSSRSSSNRTLDGATEDEALQEISGSDSVFTWQNVEYTVAYMGGERKLLNKVNGYAKPGVMVALVGASGAGKTTLLNTLSQRQKTGVVSGDMLVDGRPLGTEFQRGTGFCEQMDLHDGTATIREALEFSAILRQDRSVSRADKIAYVDKIIDLLELGDMQDALVRCLGVEQRKRVTIGVELAAKPNLLLFLDEPTSGLDSQSAYSIIYFLKKLAHAGQAIVCTIHQPSSVLIQQFDMILALNPGGNTFYFGPVGENGSSVIKYFAERGVQCPPNKNVAEFILETAAKGGKRADGKKLNWNDEWVRSKEAKQVQDEIKRINEERSKIVPPVTTDQHEFASPVTLQTVELTKRLFTQYWRDPSYLYGKLFTSVIIGIFNGFTFWKLGYSVADMQDRLFTAFLILLIPPTIVNAVVPKFYQNRALWEARELPSRIYGWVAFCTANVVAEIPIAIVGATIYWAFWYWPTGLPTDSSTAGYVYLMTVLFFFFQASWGQWICAFAPSFTVISNVLPFFFVVFSLFNGVIRPYAQLSVFWRYWMYYVNPSTYWIGGVLAATLDNTPVQCAPQEAAYFNPPPGMTCGTYAQPFVDAIKQGRLMNPGDSSNCGYCQYASGVEYLQTLNIEPRDKWRYFGIFLAFCISNWALVYFFIYTVRIRGWSFGFEYFFNLGGKGLGAVKGLFKRKGKKDMKAEA

> Phain-OT5-proseq8301

MSRAQIKTPSTFTLPPPVSPAAPPVPSTDHLDRVSYLFGHPISHSLSPLLHDTIYSALDLNYAQHLYETRSLTACLALAHSPKFFGASVTMPHKVAIIPFLDMLTPEGEAIGAVNTIFLRDHPDGRRLLCGTNTDCIGIREAILQNVRKETVSEMRGRRGMVIGGGGTCRAAVYTLKTYMGCDEVYLVNRDRSEVEQVVKECKSKGFGNGLRFVESIEEAERLQGPKVVVSAIPDIPPKSEAEIRTRKIVQTLLGKKEKGVILEMCYHPSPDTAIARISRTNEWQVIEGTEAMIWQGLEQEKVWLNKKVGALPVEKVKQVIAAKLSKPSL

> Phain-OT5-proseq8305

MAVATQLDPPSLEIDLKLDSAPQTPISRQFPRDASIVLIGSRGVGKRTLGFIGATHLGRRLITEDHYFQEITGISRGDFLRTYGNREFYKRNVEVLKRMLDQHRSGCMIECGMGSLSHQAQKALREYSKTNPVVYVTRGSERIRSLLRLGDEEAARLAAVDLSHRTCSNLEYYNLYDPSCEGIDTPPESGLGNQSSRLKYAKEDFSNFLDFLTGQGLVRNSLESPFSIAALPPEARSYTYALSLRLSTVPDLDLVELEAGADAVQLKVDTWSPDLKKMLAKQVATIRRKLGVPIIFQVEEYVFGNSPLSLPEKEHAYFQLLETGMRLGVEYIVVDLEYSCNNISQLVNTSGRTKVIGHHLLRDETSSGWDDESPMIQYRRAKSLGCDMVRFVRATSKKSDNDTVRGFLAKIESIPDHLPVIAYNLGDYGMPSLVSNRIFTPVTHPIMQATVSVSQIRRFLPTASEAMQSLYQSQILAPLHFYHLGASVFYSLSPAMHTAAYQVCGMSNDFQSLKVSSLGDIHRLCQDSNFGGAAITQPFKVQILSRIATKSYHAKAIGAANTLLPLRMLSNNNSLDGSLQSLLRQANERGKSGPIIAYYGDNTDFLGILTCLRRNISPRNVVQPSKTTGLVVGAGGMARAAVYAMIQLGCRKIFMYNRTVEHAQEVARHFNSWAAGLSSDGEIVKVLKSTSEEWPAGFTQPTIIVSCVPANSVAGEPAANFVMPMQWLASPTGGVVVELAYMPLDTPLLKQIRRFREEAKQAWIIVDGLEVLPEQAIAQFELMTGRKAPKRRMRLEVLRNYHRPFTWCPAGRVGHSLKVYDDAAV

> Phain-OT5-proseq3824

MGSSTTPDDGPTRIAILGKEDIIIDFDIWQTFVAGDLLSKLPSSTYVLITDTNIAPIYVPAFQKTFDDLTAKSDTCPRLLTYETPPGESSKGRETKAEIEDWMLSQQCTRDTVIIALGGGVIGDMIGYVAATFMRGVRFVQVPTTLLSMVDSAIGGKTAIDTPMGKNLIGAFWQPQRIYIDLRFLETLPVREFINGMAEVVKTAAIWDEAEFANLEANADLLVATIRAKSSDRSSRLEPIRDILKRIVLGSAGIKAKVVSADEREGGLRNLLNFGHSIGHAYEALLTPQVLHGEAVAIGMVKEAELARHLGVLKPGAVARLVKCISSYGLPTSLEDKRVQKLTAGKRCPVDVLLQKMAVDKKNDGKKKKIVLLSAIGKTHELKASVVEDRAIRVVLSDAIVVSPGVPKALNNEVIPPGSKSVSNRALILAALGTGPCRIKNLLHSDDTEFMLTAIAKLGGATYGWEDAGEVLLVQGKGGDLRASPTELYIGNAGTASRFLTTVVSLCKASSVKATVLTGNARMKVRPIGPLVDSLRTNGVDIEYLEKPNSLPLKVEASGGFEGGDIELAATVSSQYVSSILMCAPYAKKPVTLRLVGGKPISQPYIDMTTAMMASFGIKVIRSDTEDHTYHIPKGVYCNPAEYVVESDASSATYPLAVAAISGTTCTIPNIGSMSLQGDARFAIEVLRPMGCKVVQTDHSTTVTGPAVGQLKAIQEVDMEPMTDAFLTASVLAAVAKGTTRIRGIANQRVKECNRILAMKDQLAKFGVVCRELEDGIEVDGKPPATLKKPISGVYCYDDHRVAMSFSVLAVAAPEEVLILERECVGKTWPGWWDILSLSFGVGLSGKEVEGGHSKTASSSTAVSDKSIFIIGMRGAGKTTAGGWAAKTLNRPLLDLDVELERTTSMTIPDLIKAKGWGGFRDAELTLLKRVIDEKPKGYVFACGGGIVELRDARRLLSNYHKAGGIVLLVHRDTEQVMDYLQIDKTRPAYVEDVMGVYLRRKPWFHECSNFQYHSKSGDSGALSVAREDFGRFLSLISGQSTHFEEIRSKQHSFFVSLTMPKLSAAVGLLSAVVVGSDAVEVRVDLLEDPSSNNGIPTPEYLSVEIANLRSIVALPLIFTLRTISQGGRFPDDAQEEALGLYKAAVRMGMEYIDLEIAFPDELLQSITEAKGFSRIIASHHDPRGQLSWKNGGWMQYYNRALQYGDIIKLVGSAKSMQDNFALAQFKSAMAAAHNVPLIAINMGPLGKLSRVLNGFMTPVSHPALPFKAAPGQLSAREIRQGLSLLGEVQPKQFYLFGEPISASRSPALHNTLFQQVGLPHEYSGVETKQVEDVVELIRSRDFGGASVTIPLKLDIIPFLDDVTDAAKLIGAVNTIIPTPGQPGGPPRLLGENTDWLGMTYSLVSHSYSTSSSGAPGSALVIGAGGTARAAIYALKSLAHSPIYIVSRTPSKLASMISSFPAEFNIVPLTLSEAEALTEVPHVAIGTIPADRPIEQNMREVLAAIIRHPKADTTRQRTLLEMAYKPSTTALMQMASDAGWVTIPGLEVLSAQGWYQFQKWTGIKPLYEEARAAVLGESV

> Phain-OT5-proseq10905

MNSLVILITGASGGIGAATAIRLAHPSTSYKVKAIVLHYNSNSSKIQSIKEKIHSIDPSIKLIPIQADLSSSEDVARLHAEAVVECGPISVLFANAGTTSNASGPTGQLEDVSLETFEKTWRVNTLSSYHLTQLVVPSMLAQSFGRVIYNSSIAALTGGVVGPHYASSKSALHGMLHFLAGRYAKEGITFNAVAPALIEDTTMLPSGNDELRAKIPIGRLGRPEEVASVVELMVGNGHRRPQPVTMPTGSLYPDIDIPNVDLWTFLFERKDRPYPDNKVIYLDSDTNRSYTYAQVRAAALDFGKGLKANWGWKRGDVLALFTPNCIDTPPIMWGTHWAGGILSPANPGYTAAELSFQLKDAGAKALVTQLPFLETAREACKIAGIDEDRIILMGDDKDESNRFKHFSSFKNFSGTSRFRKAKIDPKKDLAFLVYSSGTTGHPKGVMLSHSNIVSSVLISTIGGDHNLSWTGQKDGQGDKVLAFLPFFHIYGLTVLLHQSFYNGWQLVVMQKFDLALFCSHIQKYDITFAYVVPPVVLMLGKSPIVANYDLSSLRMLSSGAAPLTRELVEAVWNRLKIPIKQGYGLSETSPTTHSQRWEDWHSTIGSVGLLLPNQTAMYMNAEDKEVPAGQTGELWIKGPNVFLGYLNNPEGTANAITSDGYFKTGDVGHQDANGNFYITDRVKELIKYKGFQVPPAELEGILTGHDDVDDVAVIGIYDESQATEVPRAYVVPKKGVEAGKAKEKEIVEWLGKKVASHKRLRGGIRFVDVVPKSASGKILRRLLKEQALKEEKDGAKAKL

> Phain-OT5-proseq6631

MLFDQGKAYAAQEEKFFDDGREEELVHFITKNADLQSSPRKILAAIDEYARTQKYLMNVGEDKGHIVSGIIRKRRPGIMLELGGYCGYSTILFADAARAAGGRKYFSLERSPKFAANIKALVEFAGLEDFVEVVVGSSNESIKRLHNNGQVKNIDMMFLDHYKPAYTTDLKLCEALGMISKNTVLAADNVIQPGNPPYLKYVRSTVQEKRMALTLASPKDTESFGLRSAAQYGSVEELGAEAKGDPDLVYRSELVNSFEPSGVPDGVEITWCLGKSEE

> Phain-OT5-proseq11138

MDPSELNIPPMKDLTIDNITENTILINSQSPDQRLTYVMERLVTHLHDFARETRLSTAEWMAALNFLVKVGQISSDVRHEFILLSDILGLSLLVDAIDHPKPASSTEGSVLGPFHTHDPPSISNGSTISSDPQGEPCLVVCTVSDQAGIPIADVKIDIWETDSTGHYDVQYSDLQSPDGRCVMKSDENGVFYFNAIVPVPYPIPHDGPVGQLLKLLKRHPWRPAHMHFMFEKEGWDHLITALYIRNDPYETSDAVFGVKQSLVVDFTTVDAATAKVYGVKEGTKLLKHDFVLIGKEETMKLRDENALKAMKKLGRRVKLIDHLPVPDLD

> Phain-OT5-proseq11232

MSRFDPNFTKHCIDTIGPKTDPRSREVLGSLIKHIHDFAREVELTPDEWMMGVKFVNSIGQASTPIRNEGQRISDVIGLESLVDEIAHAMLSDGANPTSSSILGPFWSPNAPFRELGGSIIQDPAPGGHPTLMHGKVTDLLTGKGIPNAVFDIWQASSNGKYDFQDPDNQTDNNLRGKFRTDANGEYHFYCLKPTAYSLPTDGPAGVLLKLLDRHPMRPAHIHLMISHDDFKPVTTQIFPKDDPYLTTDTVFAVKDDLVVDFLPRKDDPKAEMDLEYNVKLAPKSYTGVSPVSQTTIGLEPRL

> Phain-OT5-proseq3982

MRFSIWTLVTLLGLTLASPVPAPHLNLNPRASIEKRQTPLNAFLSLLLEYLPAVDGTLDSVLSILTTFELFLATLTGEQTTYNELGGTCTAYTVIFARGTTEPGNVGVLVGPPFFDALRTSLGASALTIQGVNDYDADIAGYEAGGDAGGSAEMAKQISATMAACPNTKLVASGYSQGGQIVHNAAAQLPAATASWISKVVVFGDPDNGTAIANVDPSKVKTFCNVGDNICVNGDLILVPHLLYGENAEEAATFVAS

> Phain-OT5-proseq4899

MTLPQPSFYLRRAGLGTTHFQYLDALEAIPFPVAMMHQPILTIILFFPLLALAFPSPESEDGAKTSLGNKSSGLLETLVGDLHAVTSKNDVVKNATTCPKMTVLFARGTAEPGNVGILTGPPFFAAIADYMNGTNQLAIQGVDYPADVSGFLVGGSVIGSAVMTALINHTLSLCPTTPLILSGYSQGAQIVHLVTSSLPSNTTSKVASVVLFGDPKNGTALQGVDARKVLTVCHAGDDICKGGDGVGAAHLNYSVDAGEAAMFVLGSAGGGLGITSRRMRTAGGGLGRAVSSYPAGPENWLE

> Phain-OT5-proseq9131

MVSFSSLVLVASAAIAGIYAAPHTSAGEVLAPRDGTPSSTGTNNGFYYSFWTDGAGDVTYSNGAAGTYTVTWSGDAGNFVAGKGWNPGAARTINFSGSYNPTGNSYLSIYGWTTNPLIEYYIVESFGTYDPSSAATQSGSVTADGSTYKILETTRTNEPSIEGTATFQQYWSVRANHRTSGSVNVTAHFEAWAALGMTLGTHNYQILATEGYHSSGTATMTVS

> Phain-OT5-proseq3700

MMKSLISFTLALALSLSPILSSVLALGQKATVTTIRPVDGDGEGLLRLAGTGLDGQILLSAEDWWGVIRAAEDLAGDFGKVTGRNLTLTNWSAINTNTTNSNSTRAAGGIRKSARSGLQSNETYTNGNGSSTTVYYTYNPVTSFVNYTIGPAATFTGPALSNAPPSQPVIITGTLHKSPLIASLIQSHKLDISSIAGRWESFISEVVTDPLPGSGIDRALVVVGSDMRGTVYGLYDVSEQIGVSPWWWWGDVPVRKREGVWALEGRKVQGEPSVRYRGVFINDEQPALTNWINSNYQPGKYGPGYNHYFYPRVFELLLRLRANYFWPAMWGSMFGVDDTANQPLADAYGIVMGSSHTEPMMRATNEWTTFGKEYGGNGQWEYDTNNASLAAFFTYGAQRAKPYVANSLFTMAMRGSGDTAITLTQAQAIAVLQDVVAKQREILGEVFNGTKMEGIPQMWCLYKEVQGYYENGLSVPDDVTLLWADDNWGNIRRLPTASETNRSGGAGVYYHFDYVGDPRDYKWINTIQLQKTVEQMQLAYARQADRIWILNVGDLKTLEIPINHFMDLAYDTPAWGYDSVPRWLGLWAEREFGPEPSVTISSVVDRYGMYAARRKYELLDASTYSIVNYNEADAILAQWQELADDAQKVYDELEDAWKPAYYEMVLQPVLGGQVVNQIYVNAGKNRLYTAQKRNTANKVAEDVLGYFKQDHVLTQRYHDLLDGKWNHMLDQTHLGYDLSYHSQNYNGYWQQPMRNTIPALSYVQELETSLAGSIGVAVEGSNATVPGDDAWHANSGSTLVLPPMSPYGPRTRWIDVFARGTQNCQWSVSISQPYVFATPNLGGTGGLNGTDTRVYVSIDWSKAPPAPSTTIVNIGINLTSSCTWGNYPAPTIQVPIHNTAVPSTFTGFVESDSHISIEAEHTTINTNTTTNTSYTTLPTHGRTLSGITLADPLASPQPPPTGPFLSYALYSFTPSPSASVTLFLSPCGNINGPDFPMEYGVAFDEEAPQVIQFIHNAASTSGSTSTAAYPLGWYQAVADGIWGSSGNTTTTVHDLSVTGRHTLKVWLVQPGVVLQKIVVDFGGGVRESYLGPPESFRAGADVVGGCCRRRRTSLWILASTLRRFFELCVILPMDPTSFIRLFLAHSLSTDAEKLPVPEVIQFCRNVREQSEATEDRLCAKLGLLPRVPQPPPQVTRSPAPTTAEPRPLRRFARGSSDSPTRRKEVKSLRWAILLVLGRNRDAEATLLDFLWFSLTVETI

> Phain-OT5-proseq2568

MRVFRTSILAASALFALAVHAVDNGLAITPQMGWDNWNAFGCSVSEELLLQTASLIVDYGLKDLGYHYVILDDCWSIGRNASSNNSLIADPEKFPNGMAAVADHLHSLGLGFGMYSDAGKYTCGMYAGSLGYETVDAQTWADWGVDYLKYDNCYNEGYAGNQQISSARYRTMGDALNATGRPILYSLCNWGEDYPWNWGSTVANSWRISGDVYDNWDTYDARCPCDGPDAWNCELPGFHCSITNIMNKASFIVSKAQPGAWNDLDMLEVGNGAMTDAEYVAHFSMWAVAKSPLIMGNDIRSIAPKDLSILSNAAVIAINQDPSGSSAARRWMYETNDDSNGKAALQMWSGSLKSTTGGDYSDMVVLLVNGANAPTVMNASLADIFVDSGPSGTAKQVKMSWEVYDLWANRMSDDEASAIIASSLDTGNATTGYNATTVGTGRYNATKTSYAEGLAQNSSLLLGNLTTTVQPSGTIVATVDPHGAALFRIRAIPTAPTDLGKRVGKDEDKNQNKGNEDVIPSL

> Phain-OT5-proseq3722

MLVSIALTALAVLPSANALVRKDGVGKLPALGWNSWNAFNCAVDEDKIMTAANEVISLGLKDVGYSYVNIDDCWSIKDARDNVTGQIVPDPVKFPSGIKGTADKIHALGLKVGIYSSAGTETCGGYPASIGVEKLDAATFASWGIDYLKYDNCYVPSNWTDQYVGCVPDGTNGQVFANGTCSISNTTAPATYDWSTSNTAERYRMMRDALLAQNRTILYSLCEWGQADVITWGNATGNSWRVTGDITPDWPRIAQILNENSFQLNYVDFWGHNDPDMLEVGNGNLTLEENRSHFAFWAAAKSPLIIGTALDLLPADLLDILKNKHLLAFSQDATFGKPATPYKWGTNPDWTFNASNPAEYWSGQSEKGTLVLALNTLDGTAQREIIWNEVPHLETKHHQRDAFEVTDIWTGEDLGCVPYGLNKVVTRDAQRKDTSLQRPSVTFDEVSAIILDPGYSSVRAGFAGEDTPKSFVNSHYGVSDAGKFTFGDDAIHNPLAGLDIRNPMSKEGIVEDWDTATQLWEYAITSRLTSFKQSDPRTNGLNDPSEIAMDVEEVEEGERTLGEHPLLMTETAWNTTKNREKSIEVAMESWGCPAFWLARNSVMAAFGGGKATSLVIDVGASTASVISIHDGLILKKSVQKSPIAGNWLSSQLRSLFATTEPKVNLVPHFAIASKTPVDAGAPAQATYRKYENKIPDSFRALEEERVLTEYKESVVQVWNGMGRLNTVNANGVSNTDYVKSQPGRVFEFPDGANQMWGVERMSVAEGLFDERAALPIPNEPALTKAQTIPEMVRQSLAAVDIDLRPHLLGNVVVTGGTTLINGFTDRLHNELTAMYPGARVKIQAAGLTAERRFGSWIGGSILGSLGTFHQMWISRKEYEEFGVNVVEKRCK

> Phain-OT5-proseq5359

MLSLLTPKQTILLVSLLSFSANASANPLRPRLDNGLALTPPMGWNTYNHYSCSPSESIVHSNAQALVDLGLQAQGYHFVTVDCGWTLPDRLANGTMTWNSTIFPSGFPALGTFLHNLGLGFGVYSDAGIQMCMTGTPAQVGSLFHEQLDATTFASWGADLLKYDNCYSEAAADYPNTDYSPTVSPSGRYQNMSSALLSTKRPIVFQICEWGVDFPSAWAPALGNSWRVTNDIIPAYRTIPRILNQVVPQTSFAGPGHWLDLDMLEVGNNVLTIPEEQTHFSFWAILKSPLVIGAALKDTTTSISTKSLNILLNKDVIGYNQDSLGVAASFRRRWTEEGYEVWAGDLSGNRMVVAVANLQDTARSLTLNLTDVGVQSVGSLKDIWNGVTASNVATAYTGHVEAHGTILLELSSFTAAAAVSAPNFYSSASFTPAGDAVRTTCSTGLCTPVGSKISYLSPTGTASLSITAASAGSKLVDVYFCNNDIAISTSWLYGTNTRNMTIAVNNVVTRIEVPLSGKSSELFSPGLGWQDTGIFKVLIGGWQEGANTVVVGNQGGAAGYQSYGADFVGMGIYS

> Phain-OT5-proseq10364

MAVLLSWLAFTLAAVSATSIVPRANSSDPLALCPGYTASNVKTTSSGLTADLSLAGAACNAYGDDLKQLTLEVVYETDDRIHVKIQDAANSVYQVPASVLPRPAASSGTDSKQTNIQFSYKESPFSFSITRGKEILFDTSAASLVFESQYLRLRTSLPENPNLYGLGEHSDPFRLNTTDYIRTLWNQDAYSIPLGANLYGAHPVYYEHRTTGSHGVFFVNSNGMDVKINNTNGKDQYLEYNTLGGILDFYFLAGPTPIDVSKQYAEVVGTPAMQAYWAFGFHNCRYGYQDAFAVAEAVYNYSRAEIPLETMWTDIDYTNFPTLAVDRRRVFSLDPDRFPLSKMQEINHYLHSHNQKQIVMVDPAVAYQDYPPYQNGAHDDIFLKRDNGSYWIGVVWPSVTVFPDWFNTGVQNYWNNEFSTFFDPSNGVDIDGLWIDMNEVSVFPCSFPCDDPYAVAVGYPPPPPDVRSPPRPLPGFSCDFQPAGTPCTGTKERRTIAAAPYSQALQVEERQAAGQELGLPGRDLLYPKYAIHNAAAFTYADNAAGGGISNHTVNTDVIHQNGLAMYDTHNLYGTMMSTASRVAMQNRRPEERPLIITRSTFAGAGTKVGHWLGDNLSDWPHYQLSIRTMIAFASIYQVPMVGSDVCGYADNTTEQLCARWATLGAFSPFYRNHNGYPPNIAQEFYQWKSVTTAAKKVIDIRYRLLDYIYTALYHQTLDGTPLINPLFYLYPQDPNTFAIENQYFYGPSLLISPVIEENSTTVSAYLPKDIFYDFYTHAPVHGQGASITINDVEITDIPLHYRGGVIVPQRVKSAMTTTELRKQDFEIVVALGSDGKASGELYLDDGISLEQKAITYLQFAFDGKKFSLKGTYGYNPGVSVARIIFLGVGSKPGGYSVNGLIAADWTHDDSTGEVVVPVGKPLTGDFEVSVN

> Phain-OT5-proseq6458

MWFPRVLAALTALLLTCNATNDGLTEAVSWDSYSLVINGNRTFIFAAEFHYQRMPVPEMWLDIFHKFKANGFNAISVYYFWSYHSPSKGVYDFETSGKNLQRLFDYAKEAGLWVIVRAGPYCNAETNGGGLALWGSDGSLGSLRTGDETYHQAWQPWVAKIGEIVAKNEITKGGTVILNQVENELQETTHSANNTLVTYMEQLETAFRAAGVTVPLTHNEKGQRSMSWSTDYQDVGGSVNVYGLDSYPGGLSCTNSDSGFNVVRNYYQWFSNYSYTQPEYFPEFEGGYFTPWGGSFYDDCAAEHDPAFPDVYYKNNIGQRTTLMSLYMAWGGTNWGHSAAPVVYTSYDYSAPLRETRQIQDKMYQTKLVALFTRVSTDLLKTEMIGNGTGYATSSTDVWTWEIRNPNTDAGFYTVQQANSSSRASVTFSADLNTSQGVITVPNINLNGRQSKILVTDYNFGQNTLLYSSADVLTYGVFYTDFLVLYLEEGQTGQFALKGTSANNTHTIFGTSEILATIEASSTTIVYTQGSGKTVLQSSGVLIYLLEQKAAWKFWAPPTTNSPDVKPGEQIFVFGPYLVRNAYVSHGVVHVSGDNDNATTIEVYTGNPDIQTIDWNGIRLDATKTLYGSVTAQIPGAEDRIVSLPALENWRDADSLPEKLNAYDDSKWTVCDKNSTLSPVAPLTKPVLFSSDYGYYMGAKVYRGYFDGNLASVNITCSGGLAFGWSAWLNGVLIGGNVGNASLATTTAVLALPISSLQLKDNVITVVVDYHGHDETSTAKGVQNPRGILGAFLISSNATNTNTNTSTIANPSTGFKLWKIQGNAGGSANLDPVRGPMNEGGLYGERTGWHLPYFRPSSPKFSRSSPLTGLNASGIQFYTTTFHLNIDSDLDVPLGLELSAPAGTVARIMIWVNGYQYGKYVPHIGPQTRFPVPPGVVNNRGLNTVALSLWAMSDEGAHLDVVRLIEYGRYQTGFRFDADWGYLQPEWDVGRLEYA

> Phain-OT5-proseq6940

MKLFRALTVAALAVQAAALSIGGKNMIVERDSDGLQDLVTYDEHSLLVYGERVYVFSGEFHPYRLPVPDLWLDVFQKIKALGFNAVSFYVHWALLEGKPGNYSAEGVFAFEPFFDAAKQAGIYLIARPGPYINAESSGGGFPGWLQRISGQLRTRAPDFLAATDNYMANIGAAIAKAQITNGGPVILVQPENEYTGSNGPVTGDFPDPVYFAYVKKQIRDSGIVVPLINNDASPKGIFAPGSVWQGSTEGDVDIYGHDSYPLGFDCANPYTWPAGSIPTTFHQTHEQESPSTFYSLDEFQGGAFDPWGGLGFAQCSVLLNMEFQRVFYKNNFASGATLLNLYMIYGGTNWGNLGHPGGYTSYDYGSSIAENREIYREKYSELKLEANFLKVSPALLTASVGTATNGTYTDSSDIFTTPLLGNGTATNFYVVRHSDYQQTTSSTYKLNLNTSQGVLSIPQLSGSLTLSGRDSKFHVTDYDLGGTALLYSTAEIFTWKKFNKQTVLVVYGGSGEQHELSVVTSSLAKQVEGTGVTSKSTNATTILNWQTSSDRQVVQVGDLLVYILDRNSAYNYWVPDFVRTDAWGAYTANIGNTTSVIVEAGYLVRSVYIEGTALHIDGDINATVPIKVIGAPANTKDLHFNSVKLDFTTDPVTGEWTSQLKYAEPKIDIPELSSLDWKYLDNLPEISSTYDDSAWKVADHNSTNNPNTLKTPTSLYSTDYGYSTGVLIYRGHFVATGNETKFDISTQGGSAFGSSVWLNSTYIGSWAGIDAASANNATYTLPNLSAGKPYIFTVVVDNQGLDENWSVGPDEMKNPRGILNYALSGHAQSDISWKLTGNLGGEDFIDKARGPLNEGGLYAERQGFTQPYPPNHNWVAGAPETGIKSAGVAFYQADFRLDLPSNYDIPLFFNFGNTTLNGSVADYRAQLWVNGWQFGKYVNNIGPQSSFPVPQGILDYHGQNWLAIELWAQQASGAQLTNFTLEAGTPVWTSIKEPELAPRPSYSKREGAY

> Phain-OT5-proseq5262

MSCLLSFALLAGAASAINLNVSATGGNASSPLTYGLMFEDINHSGDGGIYAELIQNRAFQGSLEYPSTLTPWVSIGDAVLTLQNTSVPLSKSLPTSINVASNSTNGTIGLLNPGWWGIDVKPQKYTGSFWALGSYKGKFTVKLQSDLTDEVFASLDIPSSTTPEKWAEHKFELEPRIAAPNINNTFVLEFDSKAGSLNFNLISLFPPTYNNRPNGNRPDLMEALKDLGGSFFRIPGGNNIEGDAYHYPWLWNETIGPLTERPGRPGTWGYQNTDGLGLVEYLNWCTDLSVTPVLAVWDGMYLGNEVDHILTASELEPWVQYALDELEFILGPSHSTHGALRAQLGHPEPWELKYVEIGNEDDLYDNGAETYSAYRFSMFYEAISASYPELILISSTGDYTAVGGSNTSNPSATDYHTYTRPDYFVSQFGHFDNASREHKSLIGEYACVQGNVYDQVVGVDWDAPKLPWNPWVGSVSEAIFSLGAERNGDAIIGMSYAPGFQNLNSYEWTPDLIAFTADPSQTIKSTSHHVIKLLSNNRYNATVPVTTNSDFGPAYWVAGVSEPGHYTFKAAIYNSTSTVPFSISFEGLSEGAKGTLSVLNAPDGLSSNTLENGVVSDVVKKTVTTLAAGSGGVFEFELDNYDVAVLTT

> Phain-OT5-proseq8821

MFSQSSIKRVCVFALGLIATTISPVAAGPCDIYSSGGTACIAAHSTTRALYGAYTGSLYQVKRGSDNTTTIIVPLSAGGVADAAAQDTFCASTTCLITIIYDQSGRGNHLTQAPPGGFDGPETNGYDNLASAIGAPVTLNGQKAYGVFISPGTGYRNNAVSGSATGDAAEGMYAVLDGTHYNGACCFDYGNAETNSHDTGNGHMEAIYFGDSTVWGTGAGSGPWVMADLENGLFSGESATSNTADPSISYRFVTTIVKGEPDHWAIRGANAASGSLSTYYSGVRPSVSGYNPMSKEGAIILGIGGDNSNGAQGTFYEGVMTTGYPTDATEASVQANIVAAKYVAGALTSGSALTVGSSISLRATTACCTTYYIAHTGSTVNTQVVSSSSTTALQQQASWTVRTGLGNSACFSFESVDTPGSYIRHSAFTLLLNANDGTKLFYEDATFCPQSGLNGQGTSMRSWSYPTRYIRHYSYVGYAASNGGVQSFDAPASFNDDVSFVISSGFAS

> Phain-OT5-proseq8264

MKLIYVLVALVAVAIAQTTCTLPSTYRWTSTGSLATPKSGWVSLKDFTHVLYNGQHLVYASDHDTGSSYGSMNFGLFTNWSSMASASQNTMSAATVAPTLFQFAPKSIWVLAYQWGPTTFSYKTSSDPTNTNGWSSAQTLFSGTISGSSTGAIDQTVIGDSTDMYLFFAGDNGNIYRASMPIGNFPGSFGSSSTVVLTDSTNNLFEAVQVYTVSGQNQYLMIVEAIGANGRYFRSFTATSLGGSWTPQTTTEAAPFAGKANSGATWTNDISHGDLIRSNPDQTMTVDPCNLQLLYQGKSPSSSASYDLQPYRPGLLTLQS

> Phain-OT5-proseq1022

MLRVQKITAAAIFTVTILYLLASGKVSLSPLLNPNSTAGGLFTTTPLKPTLIGAPVPLFPQPGKQTYPRAIKLRSGALLASLTVYDPENAIQLSVSHDAGTTWSPHGTVISKPAAEATALDNSFLLELPSGRILCAFRAHTKEPEALGKEEKPGGLNEGYLFFRLLVYCSDDGGKTWEYLSTGAQEPGPANGVWEPLLRLAGGQEEAGELQFFFSREMGGGRDQDNFVGSSWDGGVTWGDARTVSGAGMQTRDGMVGIQEVGGVGSGVLMAVFETVEEKGDGTVFEARFEVWSVMSRDGGVSWGERRLIYDSWFGDEGDMNRLKRNAGAPSIALVGKTLIVSFMTDEEKLEGMWHRNAYVKIITSGDGGATWGNKLIIAEKPAAWAGLLPLNATNFLVLCEHEDRVEARQVALS

> Phain-OT5-proseq8746

MIIRYSAASAFRSNGLSTVCLSCLLQTAKTKTPSRPVRRNFSIAPTRAAGVTGTTPRLRAEYFSGNTLLEKARAASRRGALTSSAPNNGSIAGQNAAAPKPEAIHTTSTSTPPPKEDLPHRKRQAARRAASQADLILPANSSSNLTSKASAAPANSLRRLVPILLSLSKPRLSILVVLTAMASYTLYPVPELLSQTETPSLSTLTLLFLTTGTTLCAASANALNMLYEPKWDAMMSRTRNRPLVRGLISTRGAVLYAVLSGVIGTTLLYFGVNPTVSFLGALNIALYAGAYTPLKRISVLNTWVGAIVGGIPPLMGWAAAAGQSATKDGTWRELLLGEGNIGGWLLAGLLVAWQFPHFMALSWSIKDEYKNAGYRMLCWVNPARNGRVALRYSLAFFPICIGLCYVGVTEWSFAIASTPINVWLVKEAVKFWRLEGIKGSAKGLFWASVWHLPVVMILAMVEKKGLWQRVWRAVVGWPDPDDEGEWMDEDEEEAVLEASKVSPEARKAAIAMPSR

> Phain-OT5-proseq2613

MLSALFSTAFLLIGFVAASTFEALHKRSIQSIDAVIDASFADPAIFEDTDGTFYTFATSHHGISVQVAMATAVNGPWEVLWHDLLPTPGNWSTGARVWAPDVRKIGENYILYYSAQNAEQTTQHCVGAATSKMVLGPYEALGTPLACPLSEGGAIDISGFTDTDGTHFVVYKVDGNSIGHGGACGNTVEPLVPTPIRLQRLEDDGYTPAGGYMDILDHIEGDGPYIEAPQIILVDGVYFLFFSSNCYATPEYDVKYATASAIRGPYTRSSNQLLQTGMPFDLTAPGGAQITRDGNFIVFHANCDNGRCMYERQIRVSGTTVTIT

> Phain-OT5-proseq10384

MVHMLKALGLGLSTVSVLVAAVPMPTAAPNILNAVALEKRASCTFTDAAAASKSKTSCATIVLNNIAVPSGTTLDLTGLTSGTKVIFEGTTTWGYEEWSGPLFSVSGENIAVSGATGNSLNGNGAKWWDGKGTNGGKTKPKFFYAHDLTGTSSITGLNILNSPVQTFSINGATGLSITDVTIDNSAGDTGSLGHNTDAFDIGSSSGITITGANVKNQDDCLAINSGTSIVFTGGTCSGGHGLSIGSVGGRSDNVVKDVTISDSTISNSANGVRIKTISGATGSVSGVTYKDITLSNISSYGIVIEQDYENGSPTGTPTTGVPITDLTISGITGSVASSATSVYILCGSSSCSGWSWSGVTFTGGKASTKCSNIPSGASC

> Phain-OT5-proseq10480

MFQLSTILSLAATAISVVSAVPVAAPEPTAAPELAKRATCTFSGSTGASAVSVSKAACATIVLSSVVVPAGETLDLTDLTSGTHVTFEGTTTFGYEEWSGPLVSVSGTSITVTGASGSLIDGDGSRWWDGEGSNGGKTKPKFFYAHSLTTSTITGLSFKDSPVQLMSINDATTLTVSGLSMDNSAGTSLGHNTDAFDVGSSTGVTITGATIVNQDDCLAINSGTDITFTSGTCTGGHGLSIGSVGGRTDNDVDSVTISSSTIKDSQNGVRIKTVYDATGTVKDVTYKDITLSGITKYGIVIEQDYENGSPTGTATTGVPITGLTVSNVKGTVTSAGTNVYILCGSGACSDWTWSGNSVTGGKTSTACSNVPSVASC

> Phain-OT5-proseq3158

MKFITRISILFVFITSTISTPWDPAPKGPANHGKTCIVQALGDQKDDTPQILKAFEECNNGGIIVFPEDQNYWIGTKLNPVIKDVTIEWKGTWTLSDNLDYWRNSSYPIAFQNHRAGFIISGERIHINGYGRGGINGNGNAWYNVEQAVTQPGRPMPFVFWNTIFVKDSPLWALNIMNGTNMWFDHITCNSTALNAPYGVNWVQNTDGFDTMDAKNIALTNMWYQGGDDCIAIKPRSYNIYVQNITCHGGNGIAIGSLGQYLEDSSVENVTVRDANIISYNNDMHNSAYIKTYVGALVPQSGYESAGLPRGGGWGVVHNILFQNFKIRGAGIGPNINQDSGNNGSFSGTSKMLVSGVSITQRRRPALPTTTSPVPQPSRNNPSLHNTMPHHAQTPTTSPSNLSPTSENRPKFDMQQRHKSSERQIFWDSFGFVPNAISCCQ

> Phain-OT5-proseq3647

MHYKLPVILAIASATLLVANPTWQAESSWIQSFEGQQAPEHTSPRPSISCFPKTPHQPLPWSPTRNKVCYVKSYNDSVTDDSTYVLNALHDCNNGGHVVFKQGLKYTIGTALDLTFLNHIDIDVQSYIQFSNDTTYWQANSFRFVFQNVTSFFKLGGNDVNIFGGGTIDGNGQIWYDLYASNIYTLRPVLFGLDGLHNSTISNLVLRYSPEYYHFIANSTNVVFDNINIAGGSKSANVAKNTDGWDTYRSSDIVIQNSHINNGDGEFLVTDLNFADIRNPDCVSFKPNSTNILIQNLFCNGSHGISVGSLGQYVGEFDIVQNIYVYNVSMHNASDAARIKVWPNTPSALSGDLQGGGGSGLVQNITYDTILVDNVDYAIEVDQCYGQSNLTLCLQYPSPLTISDVLFTNFQGKTSKKYAPETSTFACSSDSACSNIVAQGIDVQSTGGERDAFCLNVDAATLDVNCTNTYKGFN

> Phain-OT5-proseq401

MLSLFFISSILTTALAGVQLERQVYGSPCYCTNYSQIPIAVASCTEILLENIAAPNNSSIDLSKLKDNTKVTFSGLTTFGFTNSSSFEPMIFGGDNVTITAAKGAQIDGNGPAYWDGLGSNGGVPKPNHFITVSKMTGGSVIKLLHIKNWPVHLFSISGSSDLTLRDMTLDNSAGDAPNNRSNGLAASHNSDGFDVSSCNNTLIANTTVINQDDCVAVTSGNNITVDSMYCSGGHGLSIGSVGGKANNVVTNVLVRLPSSLAHVSISLINWAAKFSNSQIVNSQNGARIKTNYNTTGSIANITYSSISLTNISSYGIDVQQDYLNGGPTGDPSNGVSIENVLFRNVTGTATESAYDYYVLCGSGSCENFVFDEVEVTGGGKGASCNFPDGGCPVLK

> Phain-OT5-proseq667

MLQTTFQVLTALLLAATCIASPAPIPTAAPAPHEVEQAFKEREIEKRAATCTFSGSLGYSSASVSKASCSTIILDALTVPAGKTLDMTDLPDSTVVIFQGETSFAYSEWVGPLFAVSGTNIKVSGEASGGSILNGNGASYWDGGGGSSGVTKPKFFQAHDLTDSLIETLTILNPPVQVFSINGASNLELAYITIDGSAGDSLGKNTDGFDIGSSDTVTIGKLFQTNCNYIFVHQLKSILTKPCISENATVYNQDDCVAINSGTNIIFKNGYCSGGHGLSIGSVGGRTDNTVDGVSFLTSTVTKSVNGIRIKAIEGDTGTITDVEYDDITLSSISKYGILIEQNYDGGDLDGGTASSGVPITDLTIKNIVGTGAVSSSGYDVVITCGSSGCSSWTWSSVAVTGGKKYGSCTNVPSVAACS

> Phain-OT5-proseq9596

MKLFNTLFSIIISIRAAIASSIADASTQNLGFKTCVVNPGGNSSIDDAPAIIDAFDQCGNNGKVLFLNETYHVNTVLNTTGLKNCEVDLRGTLLWGNNITYWLNNSLPVGYQNQSSAWVFGGDNINFQGHGYGTLNGSGQAWYTFIHDQSNYPGRPHAITIANTTNSVFEGIRFVQSQMWTMTVIHSKNVLLQDIYVNNAGNDGTTSSNTDGANTIFSNNITFERWDITNGDDSIAFKANSTNIRVFDSTFHNGLGIAFGSIGQYKGEFNTIENIRVKNITCYNTLHGAYVKTWTGQQVGYPPNGGGGGLGFMKDILLSDFTLNNLSGIPFSISQCTTFSGAAGACNTSLFEIEDLTFENVAGTIGTNPIASLQCSAAAPCKNITLQNIDLALRNGTAASGYNCDAVVGSVGFNCTGSTCGTSSATGSC

> Phain-OT5-proseq11251

MLFNIFKAVVPALFLSSVAQAAAQIDTTSVGPKTALSAKSKICNVLDYGAVADNKTDIGPAILKAFSSCASTGGATIYVPPGSYSIATGVTLNKGSAYAFQIDGLITLTADGSFGGNAIVIENASDVEVFSSNGLGAINGQGYLHRISGSSQNARLFRLISCSYISIHDIIFVDSPTFHLVFNSVSNLEAYHITIRGPAIGGTDGIDLICLDNCYLHHIEVTNRDECISVKTPSNNVLIEEVYCNQSGGMSIGSLTADDVTAGDEAAVSNITMRNIYIFQCTEMLMIKTFPGGTGAVGYVKDSLFENFWAYDTTYGLDIDQYWYSHTTPDTGAVALSGLTFNNWTGTVDNGAARAPIVIRGSDIVPITDISLTNIDMWTENGNKILNQCKNVYGTGYCAGTATASPLATFTTTATTTVTPAGYTSPTSPAWGIAGYGTTLPIPVYTPAAFWSPVSSGVASASATPTS

> Phain-OT5-proseq6681

MSSPSTALFRLVAVFLFFFTLVQSASSTAEYRNVCTWGTNITYWLNNSMPMGYQNQSTAWILGGDRISFQGHGYGTIDGNGQVWYDFIKGASNYPRRPHAITVYGTTNSVFEGLRFVQSQMWTMTVIHSSNVLLQDIYVSSISNSSTSTVNTDGCDTIYANNITFHRWEVHNGDDGISPKANSTNILVTNSTFIGGAGLALGSIGQYKGVFETIENFTARDCVFINTTHSAYLKTWTGEQVGYPPNGGGGGIGCSYPPPRLPLAKGSQERKKKKNRKPINLNPDIKNVLLSAFTLSNVRQFPMSITQCINFAGAAGNCSSSLFAISNLSFLDIKGTVQQEPIIMQASKSSCSISASNLLEEQARNINNFTMPRRKIPTARANPNLKSKSESKSKSKSQVKQNRKAKTAATVMRRTTSKRFGRFRELPLEIRCMVWRFALPDPRVIEVFWNKEKGKFYTDAIQPVTLQVCKESRQETKRVFELFELKMDVQQPSFMMKWLPSQALIRYASSPAFRTYVDWSRDTLYFSTHISHVAVAHFFESLRVHPHIERKLKFMALDLSPYGASSTIRPQPKSVRAVPTIFLGIMSLKSVETIKFAFEEEREFCQPAGYNCNLSTHHRRSDQLVVNTDSKRDSLDVTKMFAIYMESLCDRIRARNQLQDFGMSEDVKPHVPTFVRVKVSREKATRYRSGPYGIERKERNADKDIEVSYIHKGMDPAQGGT

> Phain-OT5-proseq1075

MIPSPSKISTLYLITLSFLLPPATSTNTTSPSTPISTPTSTNTTTPCFCTAYPQIAPAVSACSSLTLHNIHAPAHSAIALTSLVHGTTITFSGTTTFAYTPDSQFRPIQISGSRVSIRGAPGSVIDGGGEMYWDGLGSNGGLAKPGQFMKVQITNHSTMSDVYIRNYPSHGINLAGVKDSIIQNIILNNSLGDAPNNRSNGLSAAHNSDGFNIGNSANLVLRDSKVWNQDDCVVVSDSSNVSVSGMFCSGSHGLSIAGGGSGTGHDTSNILFNDSVVTNSTNGLRIKTDFNATGSVTNVTFDNIRLEDIKKYGIDIQQDYLNGGPTGTASNGVLVRGITFRKVTGWVVEGAMDYYVLCGNGSCSDFEFKGVSVKGGNRSSCNYPETGCPGV

> Phain-OT5-proseq1809

MKLSNISTLAGIFFSSLVTAQLSGTVGPTTSTASKAATKVCNILNYGGVASTSTDNGPAILAAWTACVAGGEVYIPAGNYGMSTWVTLTGGTGVAIRLDGILYRTGTASGNMIFVEHTTDFEFFSSTSAGAMQGYGYVFHADGTYGPRLLRLAQVVGFSIHDIALVDSPAFHLTLDTCSDGEVYNMIIRGGNEGGLDGIDVWGTNIWVHDVEVTNKDECVTIKSPASYMLVENIYCNWSGGSAFGSLGADTDIHHITYDHIYTQNSNQMLLLKSNGGSGSVYSCSFTNFMGHSNAYTLDIDGYWSSESTAAGDGVLYHDLTFSHWHGTCLNGGTRAAIQALCPSGAPCYNIDIENFYIWTEAGSEVLYKDENAYGTGGGLNTGSSYTAYAVVTKTVTSVPAASYAITTMAADLTAGFAISASIPIPAVPTSFYPGLSPISAKLGCAETPGIGIHLVAHETRELCFRLAFPEHAFDDVECILGLGRRVTRTGGGRAGIGLLGRDQGWVDAPHLHAEPCDGRGDEARGAGEREGGDVF

> Phain-OT5-proseq3871

MAFVTLIARLAALTCLLYTSQAQNCWKDTACSGPDDAAFPGPWEANIYAPSSRSVSPKSILSLQTGEVISSFPGNVKLSGNGSQLVFDFGIEVGGLVHLKYLSSGPGALGLAFTEAKNWIGEWSDSSNGKLLGPDGAIYASFNESGTGTYTMPDLSLRGGFRYLTIFLVTNSTTSVEIKDIQLEIGFEPTWSNLRAYQGYFHSSDELLNKIWYSGAYTLQTNNVPPNTGRQVPFLTTGWANNGTLGPGDTIIVDGAKRDRAVWPGDMGVAVPSTFVSVGDLTAVKDALQVMYNYQNTTTGAFPEAGPPLLQLGSDTYHMWSMIGSYNYVLYTNDTAFLSQNWAKYTKAMDFIYGKVGDSGLLNVTGLRDWARWQTGFNGSEPNMILYRTLITGAELAIWAGQPSLNATWNERAVSLRTAINKYCWDSGYGAFKDNATATTLHPQDANSMAILFDVVDSPIKASSISSNLLKNWTPIGAVAPELPENISPFISSFEIQAHFSIGETARALELIRRCWGWYLNNPNGTESTVIEGYLQNGTFGYRSSRGYAYDASYISHSHGWSSGPTSGLTTYVLGLSVTGIGGKEWKLTPQFGDLTSAEGGFTTILGKYQASWRLKAGGYTLSYKVPEKTTGQIILPCLARGKWPRIEIDGHPIPRDTSPQIVGDGVVFSVAGGEHSIEVK

> Phain-OT5-proseq8712

MIFFTLIVTLAALLPRQSLVAAAGCWRDTPCTGPSAASFPGDWDQYNYSPTSRTVNPVRILSSSNTLLSEFPGPSTLSGNGSLLIFDFGKEVAGIATISYKAIGHGTLGLAFSEAKNWTGEYSDGTNGFYDFVSEGAIYGNVTATAEANYTMPIDKLRGGFRYLSVFSVADFSDGAEDKGIIEVDILDVSLEISYQPSWTNLRAYQGYFSSSDDLLNKIWYAGAYTLQTNAIPPNTGRNQVNPGWENDENLNLNTNGSTIYVDGSKRDRTVWAGDLTVALPSILISTGDLDGLKNTIQVLLNDQQSSGELPFAGPGLNIFGSDTYHMATLIGTYDYFLFTNDKDFLSANWDRYQAGMKFITDKIDSTGLLNVTGTNDWGRSASQGAHNTEANMFMYQTLTVGSALANWTGDTASAKAWSSLAATLKTAVNNLLWDGSVGAFKNSDTDASVHPEDGNSLALYFNVADASKVQSISKQLTSNWGPIGAICPELPNNIVGYIEGFEIKGHLVARQATRALDLIRRSWGWYLNNPYGTESTIIEGYLADGTFGYRADDGYNNDYAYTSHAHGWSTGPTDALTSYIVGYTLTAPGGSEWQIAPQFGDLTHAEGGFTTPLGKFAANWHTFDGGYTIAWGAPAGTSGKLVLPGANGVAPTVVKDGQIMAKEAAVYDAEAQTLTIPFQADGGAHNVNVTY

> Phain-OT5-proseq2544

MKLSLLSSGLAIFLQLASAAPTPTSDDNVVQERANIAKRATITDIATTGYATQNGGTTGGKGGTVTTVSTLAQFTTAVTNGKLTPTPLVGIGLYINKSANVIVRNIISQKVLAANGDGIGIQISTNVWVDHCELLSDLDNGKDYYDGLIDITHASEWVTVSNSYLHDHYKASLIGHSDNNGAQDTGHLHVTQHNNYWQNIGSRTPSIRFGTGHIFNSYFKNMSTGIDTRDGAEVLVQSNVFTDVDEPIAALYSDDTGYAVAIDNDLGGESNTAPAGNLTASSMPYSYSLLGSGSVVAAVVGTAGATLSF

> Phain-OT5-proseq7818

MKFLSTLLNAFILFGNASGLVAFPGAEGFGAEAVGGRTGTVYVVTNLNDSGAGSLRDAVSTEGRIVVFAVGGVIQITDRIVVPAHTTILGQTAPGGGITVYGNGWSFSNADDSIIRYIRIRMGKNGTSGKDAITLADGNTMIFDHLSVSWGRDETFSINGSGANITIQNSIIGQGLETHSCGGLIQTDGGVSLFRNLYIDNKTRNPKVKGNKVTRASSPRHAKFADDRALGVNDFTNNVVYNWGGGGGYIAGGDSNGTSYANIIGNYFISGSNTTVTAFTRGNTNFHGYVSENYYDPDQNGALDGSAIAATSSNYGGIVIGDTQYPYPAPSTILPAPQAVALAIKCGGASLVRDSIDTRLISELQTYGTVGELISDETASPMDGPGTVDPGTVALDTDGDGIPDSVEVQMGTDPEVADSMDICASGYTNIETWANSLVPSGYQY

> Phain-OT5-proseq913

MKSPTYLLVGIYTLLGQAAAVGVVGTAPGFAASTTGGGSATPQYPADIAELTAWLTDSTARVIVLDKEFNYIGSEGTVTETGCRPASNQCPGDGGQDAINHADWCTNGNAGTGVTSISVTYDAAGVTPINVGSNKSIIGIGSSGVIKGKGLRIANGAENVIIQNIHITDLNPQYIWGGDAITVAGSDLVWVDHCKISLIGRQMFVAGTTASNRVTLSNNEFDGSTSWSATCDGHHYWTIYLLGSNDLVTMKGNYIHHTSGRGPKVGSNTLLHAVNNYWYAVSGHAFDVLAGASVIAEGNVFQNVVTPLLEKVGNLFASPSTSANTACAAYLGHDCQMNSFGSSGTLSGSDTNFFSDFSGKTVATASTAGSSIQAAAGVGLI

> Phain-OT5-proseq3538

MKFSTLSASLMATLALAAPTPTVNEVADIAQIAKRASITDVGTGYASQNGGTTGGAGGTTTTVSTYAQFTAAVAGTTKKVVILSGAITETADQVKIGSNTSLIGKNSGAKLTGFGVIVKSATNVIIRNIAIAKVLADNGDAIGVQLSTNVWIDHVDVSSDQDHDKDYYDGLLDFTHAADFVTVSNSYIHDHWKASLVGHSDSNSAEDTGHLRVTYNNNYWNNINSRTPSLRFGTGHIFNSYFNEVADGINTRDGAQVLVESNTFVDSKKPLYSTDDGYAVESGNDFGTGSNEALAGTLTSVPYSYSKLGSGAVKAAVVGTAGNTLSF

> Phain-OT5-proseq10047

MFRPLQAITFLALLVFAAANPIRFPPGEFAPDECAFENDTPPPPPYAIEPTTTFIQAASSYTTPVTTASVQAASSAASSVQAASSAASSVQAASSAASSVQAASSAASSVQAASSAASSVQAASSAASSVQAASSAASSVQAASSVQTASSAAPSVQAAPSAGSSTASAPASSGFATVPASSASPAIQAASATKAVTTSSQAASSATTAVAASSQAASSATFVTTSSQAASDHCGDTDYVILTDTPWIVYNMLYNADKMVGTQCTNYQKAGTLANGDKEVAWNSVTNIEYVESTNNVPKGYSFVGLTQNLENTISSISAIPASYTWTRTNTTAFKGNVCFDFMTSDTKGDSTSSSAQELMLWLQYEGGQLPIGWGDAVATIDSLFGTSWKLYEAKNTDTGITVHSLLPDTQFEGSFNGDLKDWLEALVTIGKFTDKTYVNVGNAGTEFFYGNSILNATLPETEALLRTATALILSVDAAMDRLLSLLPTASRKAPAPALELEPQPLPDRAEDIAIQQALNEVLEYHRTHRPKNTANNYEPKQREWMAWCKKIGFKEGGKHLPEDYVDKGKLLLFIKDKVAGLRAYCSAINELWVHQISLGLYSGDRPQRVAMTALKTSIARGQHQRRRTKFEDRGLATIRDGYTALQIPDSESYVLIL

> Phain-OT5-proseq1538

MKFTQAILPFVFSAAAVLASPTPTLDKRATTICGQWDSVVTGAYTIYQDLWNEAQATSGSQCTTINSLSGSTLAWSTSWTWAGASNQVKSFANAVVSGTTKKQFSAISSIPTTWKWSYTGSNIVADVAYDIFTSSSATGSDEYEIMIWLAALGGAGPISSTGSSIATVTIDGVSFNLFSGYNGSTHVYSFVAASEATNFSGDLLKFFTYLESSQSFPSSQYLLNIGAGSEPFTGSGAVFTTSAYSAVIN

> Phain-OT5-proseq3246

MSSKLTFISLVASLLASMPVAQSSEAYPSLPTWKCTTSGGCLQQNTSVVLDQDSKYAQGAAGSRTTADYTAMGVSTSGDALTMYHYVTTNGSLNAASPRVYLLGADSKYVLLDLLNQELSVDVNYSTLPCGENGAFYLSEMAADGGGGAGAAAGTGYCDAQCPQGSCCNEMDVLEANAEATAMTPHPCDGDSCDSGGCGYNPYANGQHSFWGPGLTVDTSQPFTVTTQFAASGGTLTQISRSYIQNGRQIDGGGTISTCGTDSSSTGLTGIGNALGRGMVLAMSIWNDATQEMAWLDAGTNGPCASGAGTPSNIESQHPDTHVVFSNIRWGDIRSTATG

> Phain-OT5-proseq4170

MISTALTFSALLVSVRAQLAGTLTTETHPPLEVSQCTASGCTTSSAAIVIDANWRWLHNKVGYTNCYTGNTWNATVCPDAVTCAANCALDGADYSGTYGITTSGNSLKLSFVTKGTNTNVGSRTYLMAAGSTTAYQILSLMNQEFTFDVDVSNLPCGLNGALYFSEMDADGGMAKYPTNLAGAKYGTGYCDAQCPQDVKFINGEANSAGWTPSSNDANAGLGTYGTCCNEMDIWEANKISAAYTPHPCSVTEQTRCSGTDCGIGARYSSLCDADGCDFNSYRMGDTSFYGAGLTVDTSKPFTVVTQFISTDGTATGDLQEIKRFYVQDGTTIPNSASTVSGVTGNSITDTFCAAQKTAFGDTNEFAAKGGLKQIGAAIKKGMVLVMSIWDDHSADMLWLDAPYPPTKDASSPGVTRGTCGADSGVPATVEADSASASVTYSNIKWGPLNSTFTATAAAAGGIKNRENAKL

> Phain-OT5-proseq7132

MYRSFTLATSLVAAVVRGQQVGTLQTETHPAMTWSKCTSASSCTTQSGKVVIDSNWRWVHDKTAGSYTNCYTGNTWDATLCPDDATCAANCALEGADYATTYGATTSGNALKLTFVTKGSSATNIGSRLYLMDTDTSYQQFKLLNNEFTFDVDVSNLPCGLNGALYFVSMDADGGLAKYSTNKAGAKYGVGYCDSQCPRDLKFINGQGNVDGWKSSSNDPNAGVGGHGSCCAEMDVWEANSVSAALTPHSCSTVSQTMCDGDACGGTYSSTRYAGTCDPDGCDFNSYRQGNTTFYGKGLTVDTSKVFTVVTQFIGSPLTQIKRFYVQNGVVIPNSYSTIAGTTDYNSISTAYCDAQKAAFGDNYSFKTQGGMASMSSAMSAGMTLVMSVWDDHYANMLWLDSTYPTTDTAAGGPRGTCAVTSGVPADVEASSPGASVIYSNIKFGPIGSTFTQPAGT

> Phain-OT5-proseq6582

MFFLNRVALVAASLLAGSVNAYTSQTFTWKNVRIGGGGGFVPGIVFNPTTKGLAYARTDIGGAYRLNADDTWTPLTDFANNTNWHDWGIDALATDPVETNRLYLAVGMYTIDWDPNVGSILRSTDQGATFSETKLPFKVGGNMPGRGVGERLAVDPNKNSILYFGARSGNGLWKSTDYGVTWAKVTSFTWPGTYVQDASSEYTADPVGIAWVTFDSTSGTKGTATPRIFVGVVDVGESVFKSEDGGSTWAWVSGEPQLGFLPHKGVLSPAEKTLYISYANGAGPYDGTNGTLHKYNISTGVWTDISPTSLASTNYGYGGLSVDLQKPGTLMVAALNDWWPDEQIFRSNDSGATWSPIWAWTSYPNQDLYYSYDVSAAPWLFDTTSTDQFIKRVGWMVEALSIDPFDSNHWLYGTGATIYGGHDLLKWDTVHNVTLKSLATGIEETAALALLTPPGGPPLLSAVGDIGGFYHSSLDTAPTQAFHNPTYSTTRDLDYAGNTPADIVRSGDSDTDIKVALSSDFGATWSADYGASTSTGSGKVAYSANGDTVLLMSGTNGTLVSQYTGTFTAVSTLPSGAAIASDKRNNTVFYGGSAGSIYVSTDIAKTFAKTVALGSSTAVNQIRVNPTVAGDVWASTDTGLFHSLDYGKTFTQISTSTGPTVGYSFALGAGSTTSAYPVLYGFFTISNTTSLFKSADTGATWAIISDANHGFGAASSNVVGADISTYGRVYVGTNGRGIFYGSPS
